# Supplementary material for: Network analysis of Arabidopsis mitochondrial dynamics reveals a resolved tradeoff between physical distribution and social connectivity
Source: Cell Syst. 2021 May 19;12(5):419–431.e4. doi: 10.1016/j.cels.2021.04.006 (PMC8136767; doi:10.1016/j.cels.2021.04.006)
Supplement: Document S1. Figures S1–S6 and Tables S1 and S2 [file mmc1.pdf]

Cell Systems, Volume *12*

Supplemental information

Network analysis of *Arabidopsis* mitochondrial dynamics reveals a resolved tradeoff between physical distribution and social connectivity

Joanna M. Chustecki, Daniel J. Gibbs, George W. Bassel, and Iain G. Johnston

Network analysis of Arabidopsis mitochondrial dynamics reveals a resolved tradeoff between physical distribution and social connectivity

Combined Supplementary Information PDF

Figure S1: Networks and summary statistics from two independent videos of mitochondrial motion, related to Figures 2 and 5.

Figure S2. Physical and social statistics at different timepoints, related to Figure 5.

Figure S3. All comparisons of physical and social statistics analysed, related to Figures 5 and 6.

Figure S4. Comparison of physical and social summary statistics between experimental and theoretical networks, enforcing adjacency for encounters between mitochondria, related to Figure 5.

Figure S5. Exploration of a range of simulation parameterisations showing the range of possible behaviours of agents in the cell and networks of encounters, related to Figure 4.

Figure S6. Network efficiency of unweighted and weighted interaction networks, related to Figure 5.

Supplementary Table 1. Simulation parameterisations, related to Figures 4,5, and S2-4.

Supplementary Table 2. Simulation parameterisations for parameter sweeps, related to Figures 5 and S5.

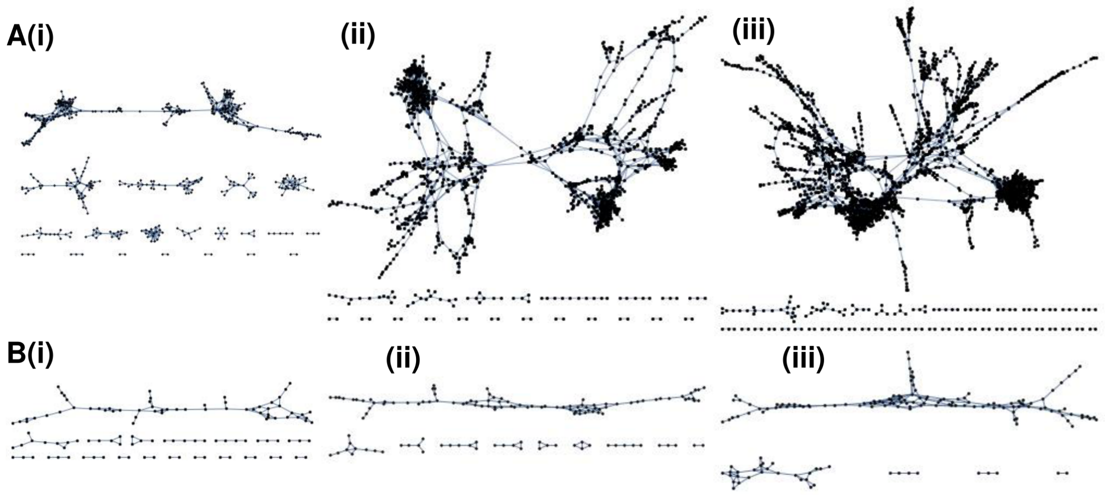

C

	logMeanDegree (at 24 seconds)	InterMitoMean (at 24 seconds)	MeanSpeed (0–24 seconds)	DegreeCV (at 24 seconds)	Network Efficiency (at 24 seconds)
Independent Video 1	1.34	1.86	4.84	1.15	0.0918
Independent Video 2	1.31	1.39	0.178	0.585	0.108

Figure S1: Networks and summary statistics from two independent videos of mitochondrial motion, related to Figures 2 and 5**.** (A) Encounter networks over time from independent video 1 and (B) independent video 2, with comparable timepoints of (i) 4 seconds; (ii) 12 seconds; (iii) 24 seconds; (C) summary statistics for both cases (singletons are omitted). Glossary: Degree, number of direct neighbours of each node; InterMitoMean, mean distance to nearest neighbouring mitochondrion (μm); MeanCH, mean convex hull area "swept out" by a trajectory (μm^2); Network Efficiency, average "closeness" (reciprocal of shortest path length) between pairs of nodes. Speed is in μm s^{-1}. See Methods (Summary statistics section) for full summary statistic definitions.

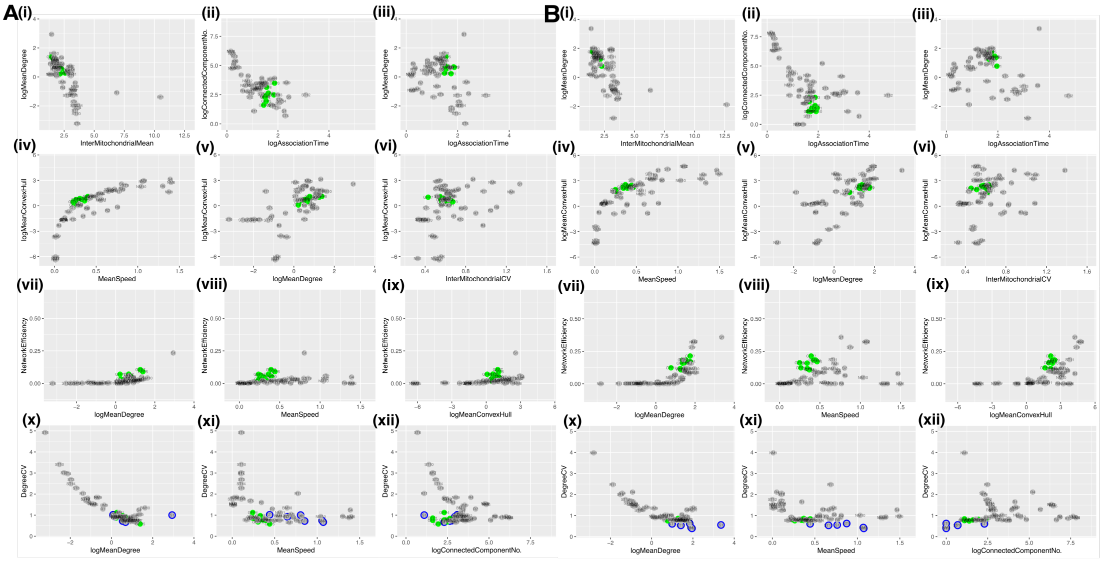

Figure S2. Physical and social statistics at different timepoints, related to Figure 5**.** (A), Statistics taken with the video end point at 1/10 total frame time (early time point, representative of 22 seconds). (B), Statistics taken with the video end point at 1/2 total frame time (early time point, representative of 115 seconds). Experimental results mtGFP ('GFP' green, n=10), theoretical results (grey, n=54), here name labels represent similarities in parameter grouping. Blue circles denote theoretical simulations of interest. CV = Coefficient of variation. Glossary: Degree, number of immediate neighbours of each node; InterMitoMean, Mean distance to nearest neighbouring mitochondrion (μm); MeanCH, mean convex hull area "swept out" by a trajectory (μm^2); Network Efficiency, average "closeness" (reciprocal of shortest path length) between pairs of nodes; Connected Components, number of connected subgraphs of the network; Association time, seconds spent in proximity to another agent/mitochondrion. Speed is in μm s^{-1}. See Methods (Summary statistics section) for full summary statistic definitions.

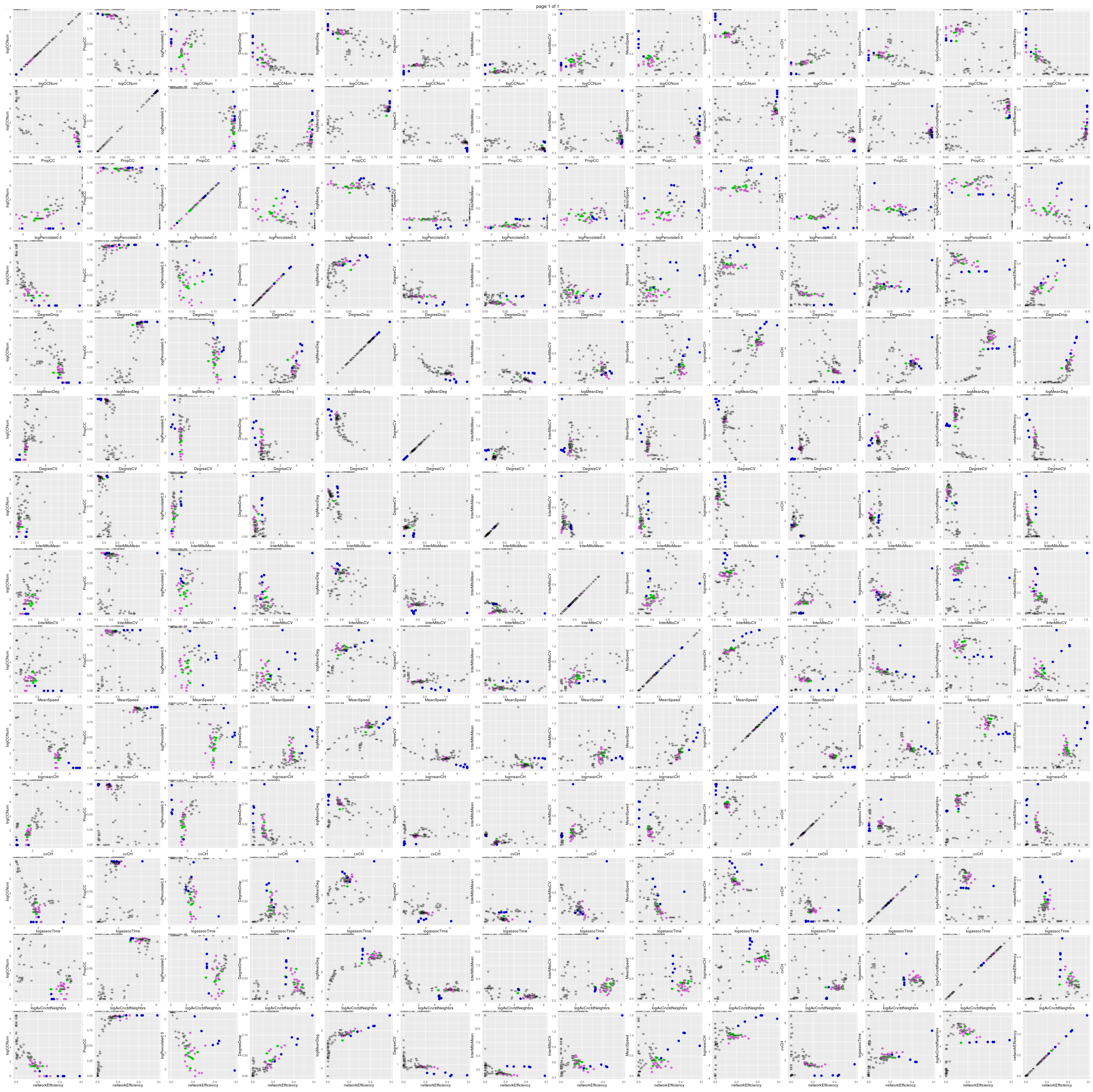

Figure S3. All comparisons of physical and social statistics analysed, related to Figures 5 and 6**.** Comparisons are taken with the video end point as the final frame (230 seconds). Experimental results mtGFP (‘GFP’ green, n=10) and *friendly* (violet, n=19), theoretical results (grey, n=54), here name labels represent similarities in parameter grouping. Blue circles denote theoretical simulations of interest. Axes label shorthand; CV = coefficient of variation, CH =Convex Hull, Deg = Degree, CC= connected component. See Methods (summary statistics and additional summary statistics sections) for full definitions.

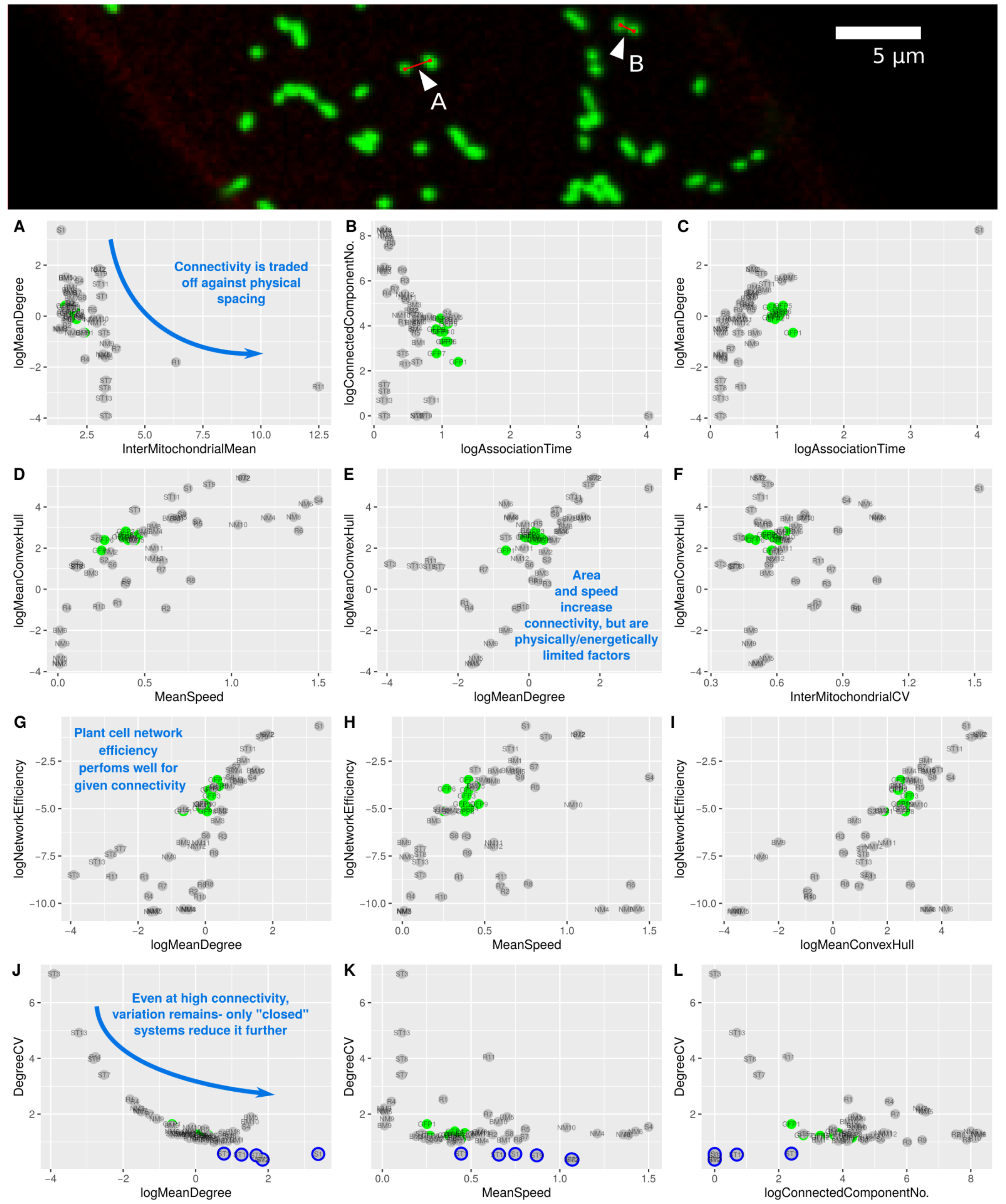

Figure S4. Comparison of physical and social summary statistics between experimental and theoretical networks, enforcing adjacency for encounters between mitochondria, related to Figure 5. Upper panel: confocal micrograph of mitochondria in *A. thaliana* hypoctyl cell (mitochondria (green), cell wall (red)) showing the original 1.6μm colocalisation distance (A, red line = 1.602μm in length), and the reduced 0.8μm colocalisation distance enforcing physical adjacency (B, red line = 0.805μm in length). Lower panels: Statistics plotted for experimental (green, *n* = 10) and simulated (grey, *n* = 47, different parameterisations) networks over 230 seconds. Labels of individual datapoints reflect individual experiments (GFP*n*, *n*=1-10, from single cell hypocotyl videos) and simulations (All described fully in Methods and full parameterisations shown in Table S1, except S5, NM1, ST4, ST6, ST10, ST12, ST14). Glossary: Degree, number of immediate neighbours of each

node; InterMitoMean, Mean distance to nearest neighbouring mitochondrion (μm); MeanCH, mean convex hull area "swept out" by a trajectory (μm^2); Network Efficiency, average "closeness" (reciprocal of shortest path length) between pairs of nodes; Connected Components, number of connected subgraphs of the network; Association time, seconds spent in proximity to another agent/mitochondrion. Speed is in $\mu m\ s^{-1}$. See Methods (Summary statistics section) for full summary statistic definitions.

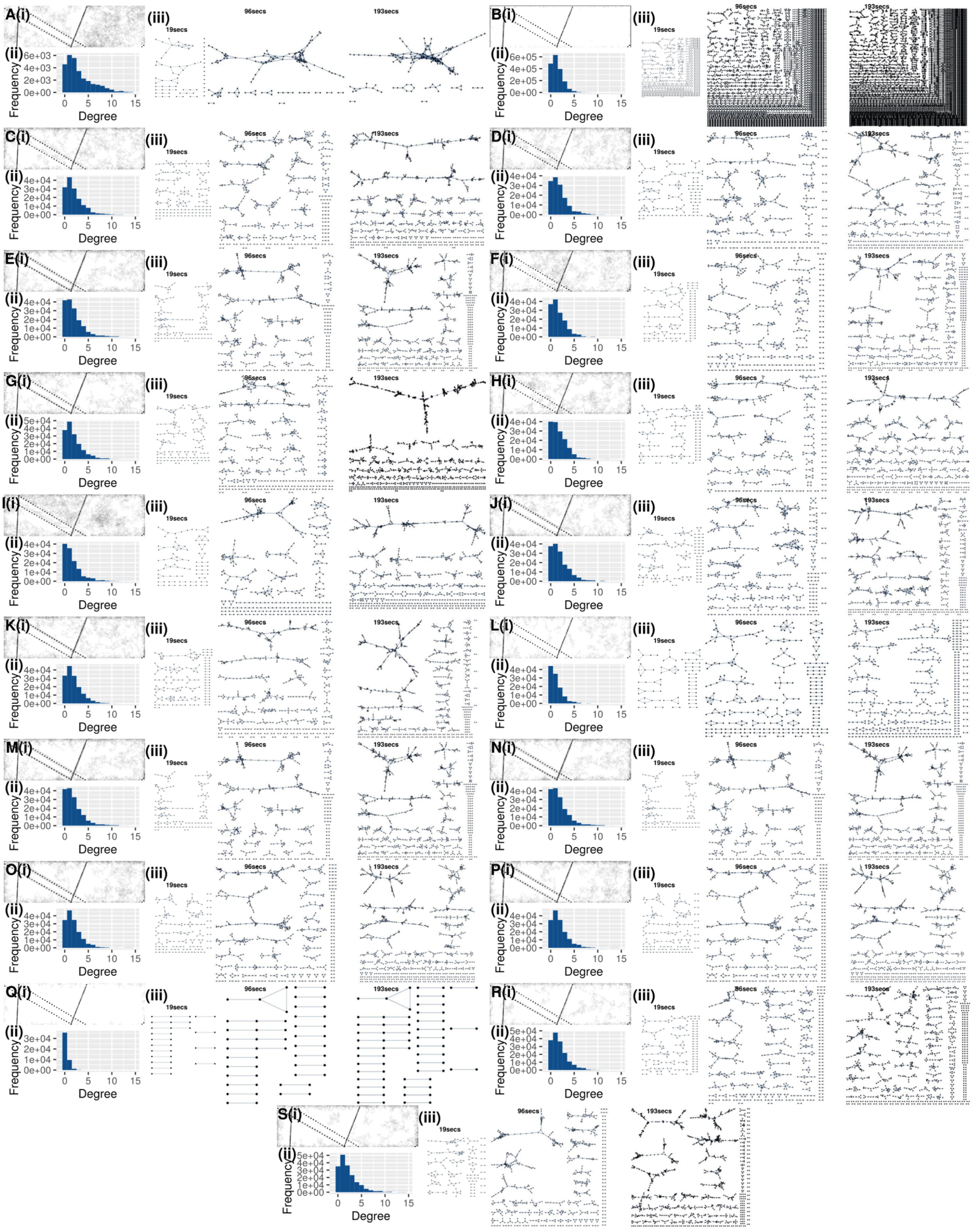

Figure S5. Exploration of a range of simulation parameterisations showing the range of possible behaviours of agents in the cell and networks of encounters, related to Figure 4. Models A-S represent 19 systems differing from an arbitrary default state**: A:** Low rate of agent loss at boundary region $K_{out} = 0.009\ s^{-1}$, **B:** High rate of agent loss at boundary region $K_{out} = 0.774\ s^{-1}$, **C:** 2:1 ratio of attachment to detachment of agents at cytoskeletal strands $K_{on} = 0.172\ s^{-1}$, $K_{off} = 0.086\ s^{-1}$, **D:** 1:2 ratio of attachment to detachment of agents at cytoskeletal strands $K_{on} = 0.086\ s^{-1}$, $K_{off} = 0.172\ s^{-1}$, **E:** 2:1 ratio of attachment to detachment of agents at cytoskeletal strands $K_{on} = 0.86\ s^{-1}$, $K_{off} = 0.43\ s^{-1}$, **F:** 1:2 ratio of attachment to detachment of agents at

cytoskeletal strands K_{on} = 0.43 s^{-1}, K_{off} = 0.86 s^{-1}, **G:** 5:1 ratio of attachment to detachment of agents at cytoskeletal strands K_{on} = 0.86 s^{-1}, K_{off} = 0.172 s^{-1}, **H:** 1:5 ratio of attachment to detachment of agents at cytoskeletal strands K_{on} = 0.172 s^{-1}, K_{off} = 0.86 s^{-1}, **I:** High hydrodynamic force F_{hydro} = 0.990 **J:** Low hydrodynamic force F_{hydro} = 0.010, **K:** Higher speed of agents on cytoskeletal strands K_{cyt} = 0.85µm/s, **L:** Lower speed of agents on cytoskeletal strands K_{cyt} = 0.009µm/s, **M:** High scaling of motion when mitochondria are interacting K_{mito}= 0.85 µm/s, but with scaling distance d_{mito} = 0µm, **N:** Low scaling of motion when mitochondria are interacting K_{mito}= 0.009 µm/s, but with interaction range between mitochondria d_{mito} = 0µm. **O:** Large interaction range d_{mito}=0.99µm, but with low scaling motion of interacting mitochondria K_{mito}= 0 µm/s, **P:** Small interaction range d_{mito}=0.01µm, but with low scaling motion of interacting mitochondria K_{mito}= 0 µm/s. **Q-S:** All have larger interaction range of d_{mito} = 5µm, and a respective K_{mito}= 0, 0.2, 0.5 µm/s scaling motion of mitochondria. For each system, we show: **(i)** Traces of paths of model mitochondria over time within simulation space. Note completely static mitochondria do not appear on traces. Where present, cytoskeletal strands are shown as black dots. **(ii)** Degree distributions of the four systems, for all nodes over whole timeframe (230 seconds). **(iii)** Mitochondrial encounter networks of these four simulated systems, at simulation times corresponding to experimentally-determined networks in Figure 2E (singletons are omitted). Full parameterisations and summary statistics of all models can be seen in Table 2.

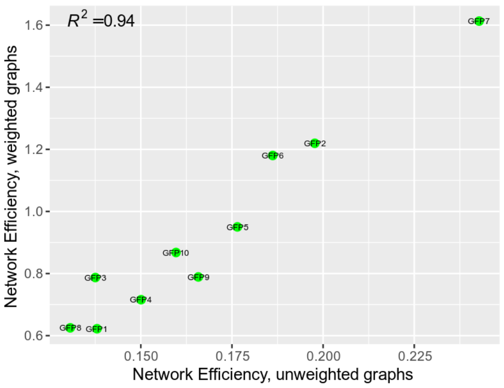

Figure S6. Network efficiency of unweighted and weighted interaction networks, related to Figure 5**.** Weighting of nodes is by 1/Association Time (secs), with the shortest distance between two nodes representative of a longer time spent associated. Data points are from the final frame of WT (GFP1-10) experimental time lapse videos. Network Efficiency; sum of all shortest routes across the network, See Methods (Summary statistics section) for full definition.

Simulation Label	N_{cyt}	N_{mito}	k_{out}	d_{cyt}	k_{on}	k_{off}	k_{cyt}	d_{mito}	k_{mito}	D	F_{hydro}
**S1**	4	128	0.000	6.008	0.809	0.479	6.412	8.339	0.770	0.754	0.000
**S2**	7	198	0.040	4.416	0.309	0.424	1.000	3.151	0.337	0.359	0.000
**S4**	16	123	0.098	4.860	0.945	0.234	1.300	0.252	0.620	0.851	0.526
**S5**	0	95	0.000	2.651	0.000	0.118	4.296	6.355	0.802	0.120	0.446
**S6**	1	154	0.050	7.079	0.000	0.706	9.341	5.627	0.408	0.431	0.000
**S7**	0	198	0.050	8.417	0.000	0.833	1.000	7.666	0.687	0.943	0.000
**S8**	12	124	0.056	1.630	0.441	0.343	5.014	3.176	0.490	0.441	0.000
**NM1**	0	100	0.000	0.000	0.000	0.000	0.000	0.000	0.000	0.010	0.000
**NM2**	0	100	0.000	0.000	0.000	0.000	0.000	0.000	0.000	0.990	0.000
**NM3**	0	100	1.000	0.000	0.000	0.000	0.000	0.000	0.000	0.010	0.000
**NM4**	0	100	1.000	0.000	0.000	0.000	0.000	0.000	0.000	0.990	0.000
**NM5**	0	100	1.000	0.000	0.000	0.000	0.000	0.000	0.000	0.010	0.990
**NM6**	0	100	1.000	0.000	0.000	0.000	0.000	0.000	0.000	0.990	0.990
**NM7**	0	100	1.000	0.000	0.000	0.000	0.000	0.000	0.000	0.010	0.010
**NM8**	0	100	1.000	0.000	0.000	0.000	0.000	0.000	0.000	0.990	0.010
**NM9**	0	100	0.100	0.000	0.000	0.000	0.000	0.000	0.000	0.010	0.000
**NM10**	0	100	0.100	0.000	0.000	0.000	0.000	0.000	0.000	0.990	0.000
**NM11**	5	100	0.100	1.000	1.000	0.500	1.000	0.000	0.000	0.174	0.000
**NM12**	0	100	0.100	0.000	0.000	0.000	0.000	0.000	0.000	0.174	0.000
**BM1**	7	198	0.040	4.416	0.309	0.424	1.000	3.151	0.337	1.000	0.000
**BM2**	7	198	0.040	4.416	0.309	0.424	1.000	3.151	0.337	0.400	0.000
**BM3**	7	198	0.040	4.416	0.309	0.424	1.000	3.151	0.337	0.200	0.000
**BM4**	7	198	0.040	4.416	0.309	0.424	1.000	3.151	0.337	0.800	0.000
**BM5**	7	198	0.040	4.416	0.309	0.424	1.000	3.151	0.337	0.600	0.800
**BM6**	7	198	0.040	4.416	0.309	0.424	1.000	3.151	0.337	0.600	0.200
**BM7**	7	198	0.040	4.416	0.309	0.424	1.000	3.151	0.337	0.600	0.000
**BM8**	7	198	0.040	4.416	0.309	0.424	1.000	3.151	0.337	0.600	0.400
**BM9**	7	198	0.040	4.416	0.309	0.424	1.000	3.151	0.337	0.000	0.000
**BM10**	7	198	0.040	4.416	0.309	0.424	1.000	3.151	0.337	0.600	0.600
**ST1**	5	100	0.000	0.000	0.000	0.000	1.000	0.000	1.000	0.400	0.000
**ST2**	5	100	0.000	0.000	0.000	0.000	1.000	0.000	1.000	1.000	0.000
**ST3**	0	100	0.000	0.000	0.000	0.000	1.000	0.000	1.000	0.100	0.000
**ST4**	15	100	0.000	0.000	0.000	0.000	1.000	0.000	1.000	0.100	0.000
**ST5**	5	100	0.000	0.000	0.000	0.000	1.000	0.000	1.000	0.200	0.000
**ST6**	20	100	0.000	0.000	0.000	0.000	1.000	0.000	1.000	0.100	0.000
**ST7**	2	100	0.000	0.000	0.000	0.000	1.000	0.000	1.000	0.100	0.000
**ST8**	6	100	0.000	0.000	0.000	0.000	1.000	0.000	1.000	0.100	0.000
**ST9**	5	100	0.000	0.000	0.000	0.000	1.000	0.000	1.000	0.800	0.000
**ST10**	4	100	0.000	0.000	0.000	0.000	1.000	0.000	1.000	0.100	0.000
**ST11**	5	100	0.000	0.000	0.000	0.000	1.000	0.000	1.000	0.600	0.000
**ST12**	5	100	0.000	0.000	0.000	0.000	1.000	0.000	1.000	0.000	0.000
**ST13**	8	100	0.000	0.000	0.000	0.000	1.000	0.000	1.000	0.100	0.000
**ST14**	10	100	0.000	0.000	0.000	0.000	1.000	0.000	1.000	0.100	0.000
**R1**	0	25	0.231	0.390	0.456	0.113	4.074	5.675	0.624	0.351	0.617
**R2**	1	92	0.585	0.363	0.685	0.040	1.945	5.062	0.417	0.820	0.537
**R3**	3	191	0.134	0.657	0.081	0.033	3.247	9.966	0.823	0.234	0.309
**R4**	2	90	0.718	0.637	0.712	0.796	9.271	5.810	0.044	0.318	0.136
**R5**	3	111	0.050	2.166	0.000	0.216	4.581	3.472	0.884	0.762	0.162
**R6**	13	159	0.565	0.796	0.985	0.366	4.181	4.870	0.879	0.413	0.642
**R7**	8	43	0.424	0.904	0.214	0.979	7.358	4.965	0.086	0.599	0.956
**R8**	3	161	0.526	0.340	0.440	0.735	3.929	0.422	0.423	0.673	0.411
**R9**	11	169	0.174	0.878	0.174	0.754	4.138	9.521	0.207	0.819	0.248
**R10**	0	183	0.745	0.313	0.259	0.964	4.211	6.884	0.571	0.177	0.609
**R11**	13	9	0.200	0.185	0.813	0.007	4.745	7.208	0.703	0.405	0.785

Supplementary Table 1. Simulation parameterisations, related to Figures 4,5, and S2-4. Simulation parameterisations used across Figure 4 and 5 and S2-4, with labels grouped by similarity. Parameter descriptions and units can be found in Materials and Methods. Rate units described here are per frame.

Simulation Label	N_{cyt}	N_{mito}	k_{out}	d_{cyt}	k_{on}	k_{off}	k_{cyt}	d_{mito}	k_{mito}	D	F_{hydro}	logMean Degree (final frame)	InterMitoMean (overall)	logMean CH (overall)	Degree CV (final frame)	Network Efficiency (final frame)
**Model A**	5	100	0.010	1.000	1.000	0.500	1.000	0.000	0.000	0.500	0.000	1.39	2.98	4.22	0.920	0.120
**Model B**	5	100	0.900	1.000	1.000	0.500	1.000	0.000	0.000	0.500	0.000	0.296	1.32	-1.73	0.905	0.000101
**Model C**	5	100	0.100	1.000	0.200	0.100	1.000	0.000	0.000	0.500	0.000	0.697	2.89	1.76	0.964	0.00773
**Model D**	5	100	0.100	1.000	0.100	0.200	1.000	0.000	0.000	0.500	0.000	0.591	2.90	1.63	0.960	0.00662
**Model E**	5	100	0.100	1.000	1.000	0.500	1.000	0.000	0.000	0.500	0.000	0.669	2.77	1.99	1.01	0.00764
**Model F**	5	100	0.100	1.000	0.500	1.000	1.000	0.000	0.000	0.500	0.000	0.537	2.91	1.60	0.948	0.00447
**Model G**	5	100	0.100	1.000	1.000	0.200	1.000	0.000	0.000	0.500	0.000	0.699	2.86	1.82	0.955	0.00780
**Model H**	5	100	0.100	1.000	0.200	1.000	1.000	0.000	0.000	0.500	0.000	0.634	2.85	1.44	0.960	0.00557
**Model I**	5	100	0.100	1.000	1.000	0.500	1.000	0.000	0.000	0.500	0.990	0.529	2.66	2.62	1.13	0.00547
**Model J**	5	100	0.100	1.000	1.000	0.500	1.000	0.000	0.000	0.500	0.010	0.694	2.85	2.03	1.00	0.00666
**Model K**	5	100	0.100	1.000	1.000	0.500	0.990	0.000	0.000	0.500	0.000	0.666	2.85	1.93	0.901	0.00705
**Model L**	5	100	0.100	1.000	1.000	0.500	0.010	0.000	0.000	0.500	0.000	0.125	2.83	1.05	1.10	0.00222
**Model M**	5	100	0.100	1.000	1.000	0.500	1.000	0.000	0.990	0.500	0.000	0.669	2.77	1.99	1.01	0.00764
**Model N**	5	100	0.100	1.000	1.000	0.500	1.000	0.000	0.010	0.500	0.000	0.669	2.77	1.99	1.01	0.00764
**Model O**	5	100	0.100	1.000	1.000	0.500	1.000	0.990	0.000	0.500	0.000	0.626	2.73	1.84	0.945	0.00511
**Model P**	5	100	0.100	1.000	1.000	0.500	1.000	0.010	0.000	0.500	0.000	0.626	2.73	1.84	0.945	0.00511
**Model Q**	5	100	0.100	1.000	1.000	0.500	1.000	5.000	0.000	0.500	0.000	-1.40	2.15	-0.0812	2.00	0.000794
**Model R**	5	100	0.100	1.000	1.000	0.500	1.000	5.000	0.200	0.500	0.000	0.654	2.40	0.669	0.944	0.00335
**Model S**	5	100	0.100	1.000	1.000	0.500	1.000	5.000	0.500	0.500	0.000	0.791	2.50	0.895	0.932	0.00548

Supplementary Table 2. Simulation parameterisations for parameter sweeps, related to Figures 5 and S5. Simulation parameterisations for model exploration and influence of individual parameters (left), alongside physical and network summary statistics (right) taken from the network of the final frame, or overall from the time series (stated) for each system. Model traces, degree distributions and networks for each model can be seen in Figure S5.
