# Supplementary material for: Network analysis of Arabidopsis mitochondrial dynamics reveals a resolved tradeoff between physical distribution and social connectivity
Source: Cell Syst. 2021 May 19;12(5):419–431.e4. doi: 10.1016/j.cels.2021.04.006 (PMC8136767; doi:10.1016/j.cels.2021.04.006)
Supplement: Document S3. Article plus supplemental information [file mmc4.pdf]

Cell Systems

Article

# Network analysis of *Arabidopsis* mitochondrial dynamics reveals a resolved tradeoff between physical distribution and social connectivity

## Graphical abstract

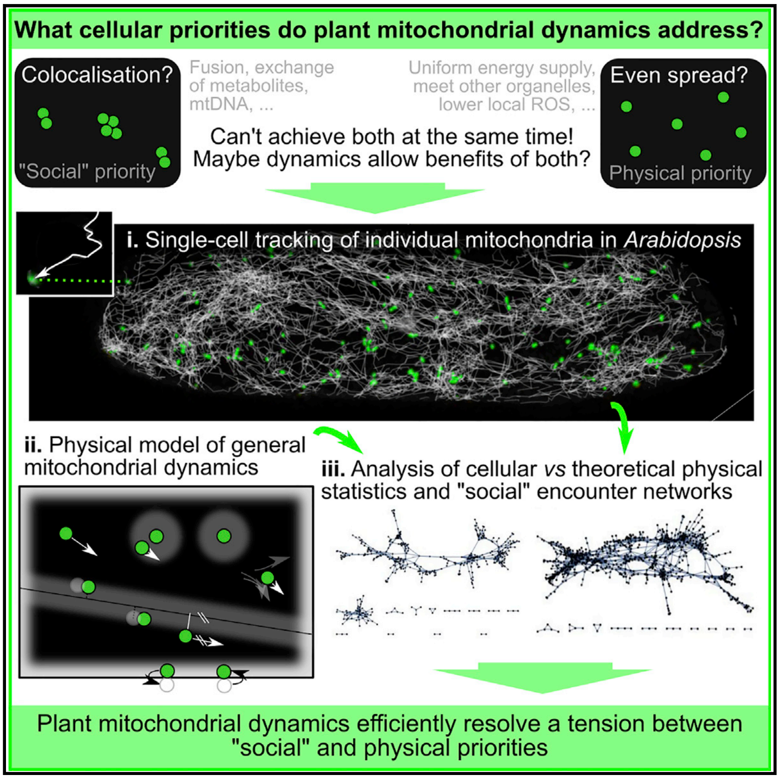


## Highlights

- Dynamic social networks of plant mitochondria reflect physical organellar encounters
- Network analysis and modeling show priorities and tradeoffs for mitochondrial motion
- Mitochondria in plant cells trade off physical spacing against social connectivity
- Plant cells favor efficient networks with high potential for information exchange

## Authors

Joanna M. Chustecki, Daniel J. Gibbs, George W. Bassel, Iain G. Johnston

## Correspondence

iain.johnston@uib.no

## In brief

Mitochondria in plant cells are highly motile and move as individuals (in contrast to the often highly connected networks in other taxa). The reasons for this motion remain unclear. This study uses single-cell live imaging of *Arabidopsis* to characterize “social” networks of encounters between mitochondria. Network analysis and modeling reveal a cellular tradeoff between maintaining the physical spacing of mitochondria and their social interactions. Mitochondrial dynamics resolve this tradeoff by providing efficient networks for the exchange of metabolic and genetic information, while keeping organelles well spread. Physical and social heterogeneity of organelles arises as a necessary result of this efficiency.

Chustecki et al., 2021, Cell Systems *12*, 419–431
May 19, 2021 © 2021 The Authors. Published by Elsevier Inc.
https://doi.org/10.1016/j.cels.2021.04.006

Cell Systems

CellPress
OPEN ACCESS

Article

# Network analysis of *Arabidopsis* mitochondrial dynamics reveals a resolved tradeoff between physical distribution and social connectivity

Joanna M. Chustecki,[1] Daniel J. Gibbs,[1] George W. Bassel,[2] and Iain G. Johnston[3,4,5,*]
[1]School of Biosciences, University of Birmingham, Birmingham B15 2TT, UK
[2]Department of Life Sciences, University of Warwick, Coventry CV4 7AL, UK
[3]Department of Mathematics, University of Bergen, Realfagbygget, Bergen 5007, Norway
[4]Computational Biology Unit, University of Bergen, Høyteknologisenteret i Bergen, Bergen 5008, Norway
[5]Lead contact
*Correspondence: iain.johnston@uib.no
https://doi.org/10.1016/j.cels.2021.04.006

## SUMMARY

Mitochondria in plant cells exist largely as individual organelles which move, colocalize, and interact, but the cellular priorities addressed by these dynamics remain incompletely understood. Here, we elucidate these principles by studying the dynamic "social networks" of mitochondria in *Arabidopsis thaliana* wildtype and mutants, describing the colocalization of individuals over time. We combine single-cell live imaging of hypocotyl mitochondrial dynamics with individual-based modeling and network analysis. We identify an inevitable tradeoff between mitochondrial physical priorities (an even cellular distribution of mitochondria) and "social" priorities (individuals interacting, to facilitate the exchange of chemicals and information). This tradeoff results in a tension between maintaining mitochondrial spacing and facilitating colocalization. We find that plant cells resolve this tension to favor efficient networks with high potential for exchanging contents. We suggest that this combination of physical modeling coupled to experimental data through network analysis can shed light on the fundamental principles underlying these complex organelle dynamics. A record of this paper's transparent peer review process is included in the supplemental information.

## INTRODUCTION

Mitochondria are key organelles in eukaryotic cells. They typically produce the majority of cellular ATP, the energy currency of the cell, and are involved in many other metabolic pathways as well as stress responses and cell death (Yoshinaga et al., 2005; Jacoby et al., 2012; Wang et al., 2018). Across eukaryotes, mitochondria take a variety of different and dynamic physical forms, from single giant reticulated networks to hundreds of individual organelles in constant motion (Jaipargas et al., 2015). The cellular principles underlying mitochondrial ultrastructure and dynamics remain poorly understood (Hoitzing et al., 2015), although roles for the exchange and complementation of biomolecules (Liu et al., 2009; Patel et al., 2013) and the facilitation of quality control (Twig et al., 2008) are likely involved.

Mitochondrial dynamics in plants differ from those in several other eukaryotes. Yeast, and often mammalian, mitochondria form elongated reticular structures, well characterized by innovative modeling and imaging approaches (Shaw and Nunnari, 2002; Sukhorukov et al., 2012; Rafelski, 2013). In contrast, plant mitochondria usually exist as separate discrete entities, presumably more resembling their alphaproteobacterium-like ancestors (Logan, 2010b). Exceptions to this rule do exist, such as in the cage-like mitochondrial formation observed in mitosis of shoot apical meristem cells (Seguí-Simarro et al., 2008), but generally, the mitochondrial population consists of dynamic, fragmented individuals, with transient fission and fusion events (Logan, 2010a). These events have been likened to kiss-and-run dynamics seen in bacterial cells (Liu et al., 2009).

The dynamic plant chondriome (a collective term for all mitochondria in the cell (Logan, 2006)) allows individual mitochondria to be effectively interconnected even without simultaneous physical linkage, with transient fission and fusion events allowing intra-mitochondrial exchange of DNA, membranes and proteins, necessary for maintaining plant health (El Zawily et al., 2014). The physics of mitochondrial motion has been characterized by elegant previous work (Logan and Leaver, 2000; Logan, 2003, 2006; Arimura et al., 2004; Scott and Logan, 2008; Arimura, 2018) using mitochondrial GFP lines, staining, and live imaging. This work has demonstrated the use of actin filaments by mitochondria as a means of transport within the plant cell (Van Gestel et al., 2002; Doniwa et al., 2007; Avisar et al., 2009; Wang and Hussey, 2015; Breuer et al., 2017) and consequent cytoplasmic streaming (Shimmen and Yokota, 2004; Ekanayake et al., 2015; Peremyslov et al., 2015; Tominaga and Ito, 2015). Logan et al. (Logan and Leaver, 2000) also highlighted the heterogeneity in mitochondrial populations, with variations in size, shape, distribution, and speed of mitochondria.


This is an open access article under the CC BY license (http://creativecommons.org/licenses/by/4.0/).

This dynamic motion of individuals, when taken together, leads to complex collective behaviors, emerging from the encounters between individual organelles (Williams and George, 2019). These collective behaviors can be viewed as reflecting the "social" dynamics of the population, where encounters between individuals constitute links in a dynamic social network. Viewing organelles as social systems, and defining organelle networks via interactions, has attracted recent interest (Cohen et al., 2018; Picard and Sandi, 2021) with most focus on analyzing the reticulated physical network structures of highly fused mitochondria (Sukhorukov et al., 2012). However, the perspective is even more apt in plant mitochondria, whose individual, fragmented nature means that social networks can genuinely correspond to the interactions of motile individuals with quasi-fixed identities, without being dictated by the physical structure of a connected network (Picard and Sandi, 2021). The mitochondrial motion in the cell leading to this collective behavior is not wholly random but is regulated and driven under cellular control (Okamoto and Shaw, 2005; Frederick and Shaw, 2007). This suggests that the emergent collective outcome of directed mitochondrial dynamics may be beneficial for the cell.

Intuitively, it is beneficial for mitochondria to be evenly spread throughout the cell, so that metabolic requirements throughout the cytosol can be met, colocalization with other organelles is facilitated, physical demands can be quickly responded to, and uneven local buildup of chemicals including reactive oxygen species is limited (Zorov et al., 2014). This intuition is substantiated by diverse lines of evidence where even distribution of mitochondria through the cytosol is compromised and the plant phenotype is observed to suffer (Feng et al., 2004; El Zawily et al., 2014). Even distribution of mitochondrial networks has also been demonstrated in yeast, with the chondriome having equal exposure to all areas of the cell (Sukhorukov et al., 2012; Rafelski, 2013; Viana et al., 2020). Further positioning of mitochondria within the cell not only depends both on cell type and structure, such as colocalization with chloroplasts in leaf cells (Islam et al., 2009), or polarized streaming in root hairs (Zheng et al., 2009) but also on energy demand (Chen and Chan, 2006; Seguí-Simarro and Staehelin, 2009; Yu et al., 2016). Alterations in mitochondrial positioning have been linked to both local energy demand and protein synthesis, showing the importance of a dynamic chondriome in the continual energy supply and regulation of metabolic demands in mammalian cells (Liesa and Shirihai, 2013; Spillane et al., 2013; Schuler et al., 2017).

In addition to even spread, interactions between individual organelles are also required for cellular function and maintaining a healthy mitochondrial population. Physical interactions of mitochondria facilitate the beneficial exchange of genetic material, protein machinery, and other biomolecules between organelles (Arimura et al., 2004; Logan, 2006; Takanashi et al., 2006; Arimura, 2018): exchange of mitochondrial content has been measured using photoconvertible probes, with mixing throughout the entire cellular population occurring over 1–2 h (Arimura et al., 2004). Spatial proximity is a necessity for this fusion, and the exchange of content (occurring within 3 s) is preceded by a period of direct adjacency between mitochondria (~20 s) (Arimura et al., 2004). This exchange helps compensate for damaged material in individual organelles and improves the performance of the cellular population (Karbowski and Youle, 2003; Twig et al., 2008; Figge et al., 2012; Shutt and McBride, 2013). Proximity, though not necessarily direct adjacency, between organelles also facilitates functional metabolic effects (Jones, 1986), including the modulation of glucose concentration (as analyzed by (Agrawal et al., 2018) in axons) and facilitation of metabolite control and flow between organelles as in photorespiration (Bauwe et al., 2010; Shai et al., 2016) and C4 photosynthesis (Sage et al., 2012).

Following these observations, we hypothesize that the plant cell has dual (and conflicting) priorities shaping mitochondrial behavior: the even spread of mitochondria throughout the cell and the facilitation of "social connections"—physical encounters between pairs of organelles that together build a network of encounters through the whole organelle population. Resolutions to this conflict would offer both a chondriome responsive to fluctuations in cellular energy demands and avoidance of local oxidative stress and an interconnected dynamic population of organelles capable of facilitating inter-mitochondrial exchange, complementation, and metabolic interaction.

Here, we embrace the emerging perspective of inter-organelle interactions in cell biology (Valm et al., 2017; Cohen et al., 2018; Picard and Sandi, 2021) and use a combination of single-cell microscopy with physical and network analysis to explore this hypothesis, seeking to identify the mechanisms and governing principles underlying the physical and social dynamics and heterogeneity in plant mitochondria.

## RESULTS

### Characterizing the single-cell "social networks" of mitochondria in *Arabidopsis* hypocotyl

To compare hypothesized mechanisms shaping mitochondrial dynamics, we first sought to experimentally characterize the collective behavior of cellular populations of mitochondria. We use laser scanning confocal microscopy to record mitochondrial dynamics in a quasi-flat plane of the cytosol in single hypocotyl cells of 5-day *Arabidopsis* seedlings encoding mitochondrial-targeted GFP (mtGFP) (kindly provided by Prof. David Logan, Logan and Leaver, 2000) (STAR methods; Figure 1A). One representative video is shown in Video S1; all others are available online (see resource availability). These cells are roughly cuboidal, and the presence of the central vacuole means that the cytosol forms relatively thin sections around the quasi-planar cell surfaces, making these 2D slices representative of a subset of the cytosol. The mitochondria captured within each video were tracked and quantified using TrackMate (Tinevez et al., 2017), building a set of manually verified physical trajectories over time (Figure 1B).

To translate these physical dynamics into dynamic "social networks," we defined mitochondrial encounters as pairwise colocalization within a threshold distance (see STAR methods). Throughout, we did not insist on encounters being defined via fusion events. These events are rather rare, and a characterization of the corresponding network of interactions would have required imaging over timescales that would stress the cell. We rather focus on colocalizations within a threshold distance, a necessary prerequisite for fusion, which provides the physical foundation for the exchange of organelle contents (and which itself has functional consequences). Colocalizations are more

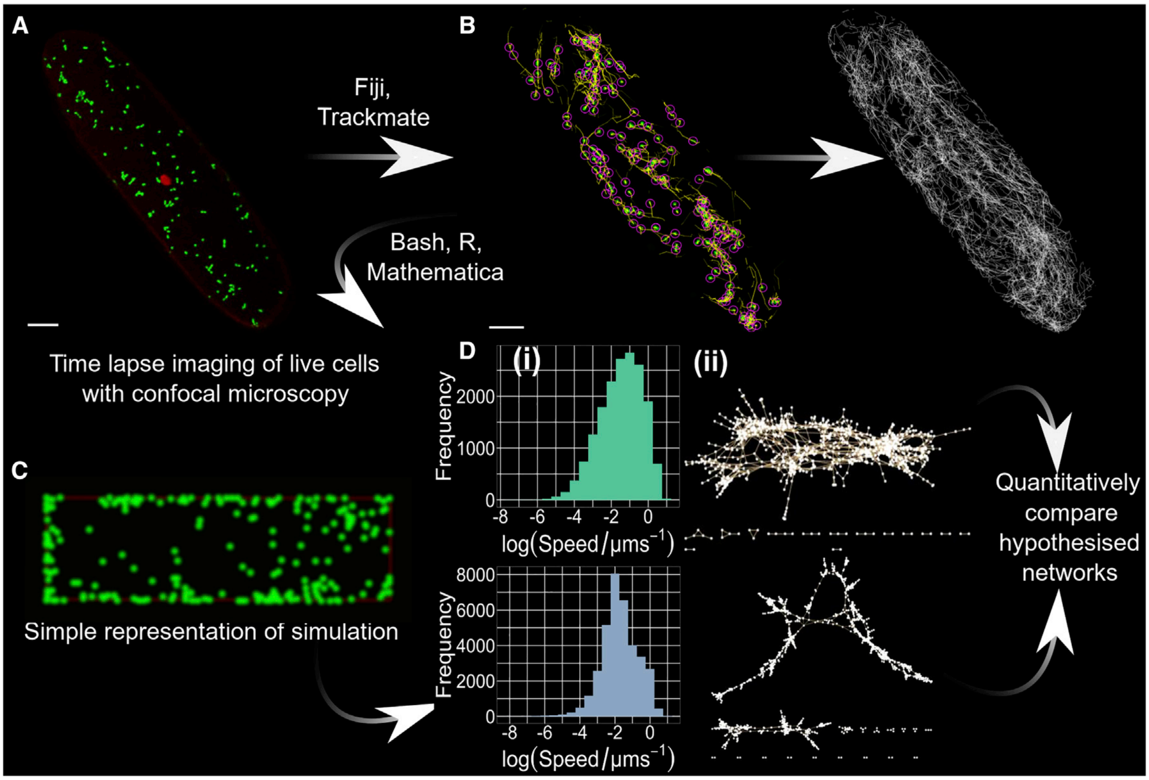


**Figure 1. Characterizing the "social networks" of mitochondria in plant cells**

(A) Single-cell video acquisition. One frame of mitochondria (green) in mtGFP *A. thaliana* hypocotyl, cropped to one cell. Chloroplast autofluorescence is also captured (red). Scale bar, 10 μm.

(B) Establishing mitochondrial trajectories. Traces (yellow) of mitochondria (green) tracked with TrackMate software. For clarity, yellow traces (left) are limited to ten frames; white traces (right) are not truncated. Pink circles demonstrate the result of computational spot detection identifying individual mitochondria—a diameter of 1 μm was used in the analysis, but a 2.5-μm diameter is shown here for clarity. Scale bar, 10 μm.

(C) Individual-based modeling of control mechanisms. *In silico* simulation of dynamic mitochondria following different rules (snapshot shows mitochondria in green) are used to generate trajectories for the same analysis in (D).

(D) Physical and social summary statistics. (Di) Physical statistics such as log(speed/$\mu ms^{-1}$) are collected directly from trajectories, here showing all speeds over all frame times. (Dii) Colocalization analysis is used to construct dynamic networks describing organellar encounters (mitochondria are nodes, colocalizations are edges). Summary statistics of these networks are then computed. See also Video S1.

frequent, can be measured less ambiguously, and provide a powerful means of analyzing the physical-social dynamics of the chondriome independent of their functional effects. To test the robustness of our findings to different and stricter definitions of organelle encounters, we explored different values of this thresholding distance, including enforcing an "encounter" to involve directly adjoining mitochondria, and found that our results remained robust to this more stringent colocalization requirement (Figure S4).

Networks of mitochondrial encounters were built up from these colocalization events, with each node representing a mitochondrion and each edge representing a current or previous colocalization event. These networks are dynamic, with new encounters occurring and mitochondria entering and leaving the system over time (see STAR methods section for discussion of timescales). As described below and in STAR methods, we also ran physical simulations of model mitochondrial populations to explore the influence of different control mechanisms (Figure 1C) and tracked organelles and built model encounter networks in the same way. From both experimental and simulation observations, these coupled measurements allow quantification of the physical (Figure 1Di) and social (Figure 1Dii) behavior of organelles within the cell and comparison with mechanistic models. The same method was applied to previously published videos of mitochondrial motion in order to independently verify this approach, yielding quantitatively similar network structures and statistics (Figure S1).

## Network analysis of mitochondrial interactions reveals social heterogeneity paralleling physical heterogeneity

The physical statistics collected from our mtGFP cells confirm previously reported cellular heterogeneity in mitochondrial motion (Logan and Leaver, 2000; Arimura et al., 2004) (Figures 2A–2D). To illustrate typical features of this mitochondrial motion, in Figure 2, we analyze a representative biological instance of a representative mitochondrial population (we will analyze a larger collection of cells in subsequent sections). This sample is also seen in Figures 1A, 1B, 1Di, and 1Dii and Video S1. Both ballistic and diffusive mitochondrial motion, with speeds spanning at least an order of magnitude, was observed (Figures

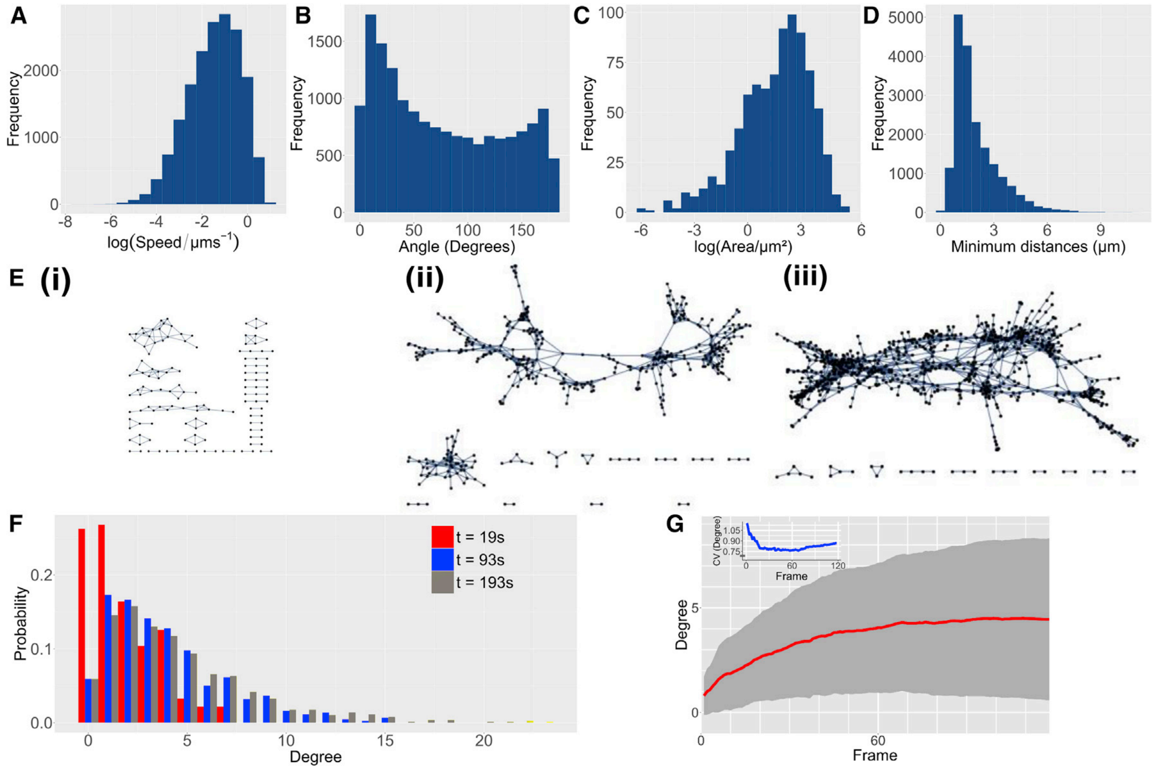


**Figure 2. Physical and social mitochondrial heterogeneity within a representative mtGFP hypocotyl cell of *A. thaliana***

(A–D) Physical statistics gathered from a single cell population of mitochondria moving for 230 s: (A) Instantaneous mitochondrial speed.

(B) Angle of motion across triplets of frames (0 deg = constant, 180 deg = reversal).

(C) Convex hull area "swept out" by a mitochondrion.

(D) Distance to a mitochondrion's nearest physical neighbor.

(E) (Ei–Eiii) Social" networks of mitochondrial encounters (nodes, mitochondria; edges, interactions) showing all encounters recorded up to times 19, 96, 193 s, respectively. Singletons (mitochondria with no interactions) are omitted from network visualization for clarity.

(F) Corresponding degree (number of immediate neighbors of each node) distributions of networks (Ei–Eiii) (red, 19 s; blue, 96 s; gray, 193 s).

(G) Time series of mean (red), standard deviation (gray region), and coefficient of variation (inset, blue) of degree for nodes in the encounter networks built up during the duration of the video. See also Figure S1.

2A and 2B). The areas of convex hulls of mitochondrial trajectories (a measure of the area covered by a mitochondrion over time) were also highly heterogeneous (Figure 2C), with many mitochondria covering a small area and fewer covering a large area. Minimum physical distances to a mitochondrion's nearest neighbor also varied dramatically across organelles, with a mean of 2.00 μm taken across organelles within a single cell, and a coefficient of variation (CV) of 0.64 (Figure 2D).

Our analysis of mitochondrial interaction networks showed that this physical heterogeneity is mirrored by social heterogeneity. An example of an encounter network evolving over time is given in Figure 2E, where several general principles can be observed. Over time, the number of social connections between individuals (edges in the network) increases as more mitochondrial pairs colocalize, and the number of organelles with small "social groups" correspondingly decreases. Large sub-networks grow and coalesce, as bridging connections form between organelle partners. The network structures, which we explore in quantitative depth later, contain several loops and strands, where denser collections of more clique-like organelles are linked by a limited number of itinerant mitochondria passing between groups.

Degree centrality, a measure of the number of partners a mitochondrion has encountered over time, follows highly skewed distributions (Figure 2F), with CV approaching 1 in some cases (Figure 2G). This situation corresponds to many mitochondria encountering few partners, but some mitochondria encountering many partners (reminiscent of hubs in human social networks). These high degree nodes are indeed observed to be the most itinerant of the mitochondria in the cell (confirmed by later analysis, for example, in Figure 5E). The scale of these degree distributions expands over time as more encounters occur, with mean degree increasing with sigmoidal dynamics, and degree CV also increasing after a transient decrease due to early interactions between previously isolated mitochondria (Figure 2G). The cellular population of mitochondria, thus, experiences a diverse range of social connectivity over a wide range of time scales.

## Individual-based modeling identifies network signatures of different cellular control mechanisms

We next sought to use these combined physical and social statistics to compare hypotheses for the mechanisms underlying mitochondrial motion. To this end, we built a predictive

CellPress
OPEN ACCESS

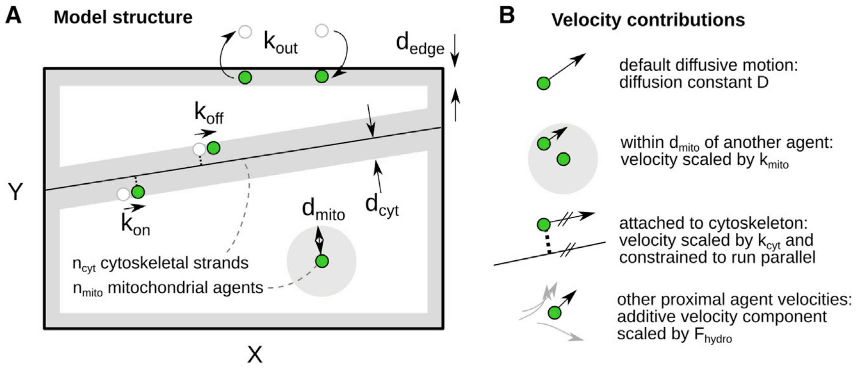


**Figure 3. Umbrella model capturing different physical mechanisms for mitochondrial dynamics**

(A) Structural features of the model. An X × Y cell contains $n_{cyt}$ randomly placed, linear cytoskeletal strands, and $n_{mito}$ mitochondrial agents. Within a distance $d_{edge}$ of the cell boundary, agents are removed with rate $k_{out}$, to be replaced by a new agent randomly positioned in the cell boundary (modeling motion into and out of the plane). Within a distance $d_{cyt}$ of a cytoskeletal strand, unattached agents attach with rate $k_{on}$ and attached agents detach with rate $k_{off}$.

(B) Features influencing mitochondrial motion. By default, motion is diffusive with diffusion constant D. When within a distance $d_{mito}$ of another agent, an agent's instantaneous velocity is scaled by a factor $k_{mito}$ ($k_{mito}$ = 1: no effect). When attached to the cytoskeleton, an agent's velocity is scaled by $k_{cyt}$ and constrained to run parallel to the strand. Cytoplasmic streaming is modeling through an additive contribution from all other agents' velocities, scaled by distance and a parameter $F_{hydro}$ ($F_{hydro}$ = 0: no effect).

individual-based computer model of mitochondrial motion in the cell. This "umbrella" model captures a range of possible mechanisms shaping mitochondrial motion. Different parameterizations can be used to assign different rates and magnitudes of influence to each mechanism (or to remove some completely). This *in silico* framework allows us to impose different mechanisms and constraints on the model system (Figure 3), to uncover which physical principles hold regardless of control mechanism, and also to compare model behaviors to experiments, to uncover which of the range of physical possible behaviors resemble biological reality. Observations and measurements of mitochondria in plant hypocotyl cells were used to parameterize the basic size, shape, and number of mitochondria in the system (see STAR methods). Other parameters control the relative strengths of different influences in the cell and can be varied arbitrarily allowing us to explore the dynamics, control, and potential tradeoffs of the system. We note here that we are not explicitly solving an inference problem, nor attempting to learn sets of parameters that best describe our biological system. Instead, we aim to characterize the range of behaviors that are physically possible in a population of plant mitochondria. This range will inform about the theoretical limitations of the system: for example, following our hypothesis, is it indeed challenging for the model under any parameterization to achieve both even spread and regular encounters between mitochondria? We will then compare experimentally observed behaviors to this comprehensive space of potential physical behaviors to identify which resolutions to these tensions may have evolved in nature.

Following our initial hypothesis that a combination of [i] even physical spread and [ii] regular social encounters is beneficial for the cell, we generated four illustrative simulations (Figures 4A–4D; parameterization given in Table S1 [NM9-12]). Systems B and D constitute "null models" for mitochondrial motion, where motion is dominated by purely random diffusion. System (A) has a low diffusion constant and no cytoskeletal strands, resulting in most individuals being evenly spaced (achieving [i]), but relatively static (failing to achieve [iii]). This is reflected in the low degree values for nodes of networks from (A) across time and their average (Figures 4Aii and 4E). System (B) has a high diffusion constant and no cytoskeletal strands. In the traces of paths traveled by agents in the system, we see a dense covering of simulation space, with individuals moving rapidly, with the opportunity to meet with many others (Figure 4Bi). This is reflected in network statistics including the degree distribution (number of encounters for each mitochondrion, Figure 4Bii) and the larger proportional size of the largest connected component (the largest fraction of mitochondria that can be linked via encounters, Figure 4Biii). In principle, this increased encounter rate provides a means to jointly achieve even spread and regular encounters. However, the diffusion rate necessary to achieve this balance (0.85$\mu m^2$/s, system [B]) is much higher than the maximum diffusion rate we observed (0.15$\mu m^2$/s, see STAR methods) and that observed in other studies (0.091 $\mu m^2$/s, Zheng et al., 2009), suggesting that this approach is either physically inaccessible to plant cells, or that it has other detrimental consequences and hence is not employed in nature.

When diffusion rate is limited to biologically reasonable values, system (C) also implements motion on cytoskeletal strands and is successful in facilitating encounters between agents. This is partly due to the cytoskeletal strands offering routes of transfer from one cell area to another, allowing separate network components (different mitochondrial "social circles") to connect. When contrasted to system (D), of the same diffusion rate but without cytoskeletal strands, we see the success of cytoskeletal strands facilitating encounters more clearly. The quasi-1D motion along cytoskeletal strands can be pictured as reducing the effective space in which mitochondrial agents move, increasing encounter probabilities and density and leads to higher magnitude displacement than the 2D diffusion case. Correspondingly, mean degree is higher in system (C) than (D), demonstrating increasing network connectivity and a decreased inter-mitochondrial distance (Figure 4E). These differences are a result of the increased area traversed by organelles once cytoskeletal strands are introduced into the simulation (Figure 4E).

Further, this increased connectivity facilitates an increase in the efficiency with which information and content can potentially be exchanged through the population. We measure this with "network efficiency," which reports how close the social connection between pairs of mitochondria is, averaged over the network. A lower efficiency corresponds to more difficulty in sharing content through the population; high efficiency corresponds to short paths between many pairs, allowing fast sharing.

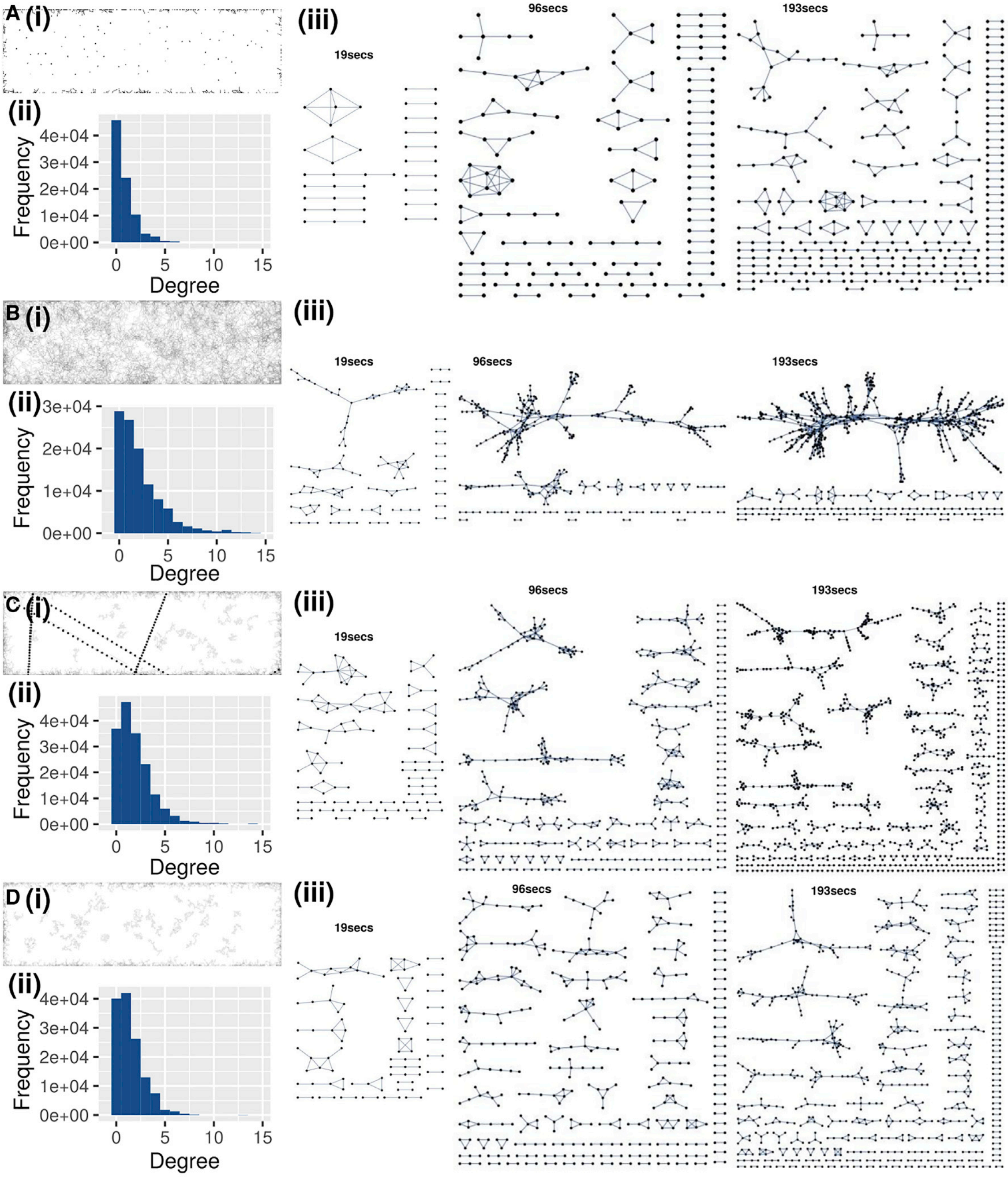


**E**

| | logMeanDegree (final frame) | InterMitoMean (overall) | logMeanCH (overall) | DegreeCV (final frame) | Network Efficiency (final frame) |
|---|---|---|---|---|---|
| *network (A)* | −0.161 | 3.36 | −2.64 | 1.27 | 0.00161 |
| *network (B)* | 0.755 | 3.04 | 3.14 | 1.22 | 0.0465 |
| *network (C)* | 0.674 | 2.64 | −0.00767 | 0.936 | 0.00393 |
| *network (D)* | 0.414 | 2.92 | −0.108 | 0.975 | 0.00289 |

**Figure 4. Individual-based model characterizes mitochondrial behaviors under different cellular mechanisms**

Four systems illustrate different behaviors:

(A) Low diffusion rate D = 0.009 $\mu m^2/s$ and edge disappearance probability ($k_{out}$) = 0.086 $s^{-1}$.

(B) Higher diffusion rate D = 0.85 $\mu m^2/s$ and $k_{out}$ = 0.086 $s^{-1}$.

(C) Intermediate diffusion rate D = 0.15 $\mu m^2/s$, $k_{out}$ = 0.086 $s^{-1}$, (5 strands, $d_{cyt}$ = 0.85 $\mu m$, $k_{on}$ = 0.85 $s^{-1}$, $k_{off}$ = 0.43 $s^{-1}$, $k_{cyt}$ = 0.85 $\mu m/s$).

*(legend continued on next page)*

We observe a quantitative increase in network efficiency in (C) compared with (D), showing that motion on cytoskeletal strands substantially enhances network connectivity and sharing efficiency, versus purely diffusive motion.

Following this analysis of the main tradeoffs revealed by the physical model, we further characterize its behavior under different parameterizations in Figure S5. Briefly, a wide range of reasonable fluxes of mitochondria into and out of the cellular subregion ($k_{out}$) preserved broad model behavior; a higher $k_{on}$ : $k_{off}$ ratio led to more motility, connectivity, and network efficiency; absolute values of $k_{on}$ and $k_{off}$ had less impact on dynamics than their ratio. Other parameters had more limited effects, although high values of our hydrodynamic interaction parameter $F_{hydro}$ led to less ballistic motion and more aligned mitochondrial trajectories (Figure S5).

## *Arabidopsis* mitochondrial dynamics are characteristic of a tradeoff between physical and social priorities

Having established an *in silico* model for mitochondrial dynamics in our system, we next asked to what extent our hypothesized tension between physical spread and social encounters of organelles exists in theory (Logan, 2007) and how real plant cells might resolve this tension through control of mitochondrial dynamics. To this end, we simulated a wide range of different behaviors in our physical model and observed the corresponding range of behaviors, including the performance of different mechanisms with respect to this tension (Figures 5, S2, and S3; STAR methods [section: Model behavior under different parameterizations]).

Theory suggests that a tradeoff indeed exists between even spread and social network connectivity, revealed as a negative correlation between (physical) inter-mitochondrial distance and (social) mean degree (Figure 5A). Comparison of the theoretically possible range of behaviors with real experimental observations show that, faced with this tension, plant cells adopt dynamics that allow high social connectivity, at the expense of even spread (Figure 5A). More specifically, plant mitochondria maintain a moderate association time when they meet, which is shown by theory to allow the dynamic formation of a well-connected network with fewer, larger connected components and high degree (Figures 5B and 5C).

The remarkable mitochondrial motion in plant cells then emerges as a compensatory mechanism, allowing mitochondria to achieve what physical spread they can while adopting this high-connectivity poise. Intuitively, an increase in speed allows larger areas in the cell to be traveled (Figure 5D), which allows a higher social connectivity (Figure 5E). This increase in degree through the area traveled illustrates the role of itinerant mitochondria and their coverage of the cell contributing to global connectivity across the mitochondrial population. However, in real cells, mitochondria cannot achieve arbitrarily high speeds (due to physical and energetic constraints) and so cannot make unconstrained use of this compensatory mechanism (Figure 5E). The mitochondrial dynamics we and others observe can thus be viewed as a means to facilitate the spread of organelles through the cell while allowing regular encounters and while limited by physical constraints.

Theory shows that the heterogeneity we observe in inter-mitochondrial distance is an inevitable consequence of resolving this tension in these complex, multi-agent systems. Simulations show that this heterogeneity cannot be reduced much further from its experimentally observed values without limiting the area that mitochondria cover, and hence without limiting connectivity, which is modulated by the area covered (Figures 5E and 5F). This suggests that physical heterogeneity is an inevitable consequence of achieving high "social" connectivity.

Notably, for the level of connectivity observed in their encounter networks, the network efficiency observed across plant cells is close to the maximum observed theoretically (Figure 5G). As above, network efficiency measures the accessibility of one node from another in a network (see STAR methods); it is higher if paths between nodes are shorter, and so, here, can be facilitated by denser contact networks. Efficiency dictates the ability of the mitochondrial population to share content between individuals: for example, the potential exchange of genetic information and protein machinery (Arrieta-Montiel et al., 2009; Mouli et al., 2009; Hyde et al., 2010; Patel et al., 2013; El Zawily et al., 2014; Welchen et al., 2014). The observed high network efficiencies are thus desirable for the maintenance of chondriome function through complementation of damaged genomes and proteins (Logan, 2010b). To test whether network efficiencies were influenced by the length of association time between mitochondria, we used reciprocal association time (in seconds) to weight edges in the experimental networks and recomputed efficiency with these weighted edges. A tight correlation exists between network efficiencies of unweighted and weighted graphs (Figure S6), demonstrating the ability of the unweighted networks to sufficiently capture underlying behaviors and resulting network efficiencies.

For a given speed and area traveled by mitochondria, unweighted plant cell encounter networks show strikingly high network efficiencies, approaching and sometimes exceeding the predictions of the theoretical model (Figures 5H and 5I). This suggests that the dynamics of plant mitochondria successfully optimizes the ability to transfer information through the population of mitochondria.

We also found that the resolutions adopted by plant cells to the core tradeoff above entails an unavoidable level of heterogeneity in the social connectivity of mitochondria. Our model suggests a reasonably tight negative relationship between degree coefficient of variation (CV) and mean degree (Figure 5J) – the less connected

(D) Intermediate diffusion rate D = 0.15 $\mu m^2/s$, $k_{out}$ = 0.086 $s^{-1}$, no cytoskeletal motion. For each system, we show: (Di) Traces of paths of model mitochondria over time within simulation space. Where present, cytoskeletal strands are shown as black dots. (Dii) Degree distributions (number of immediate neighbors of each node) of the four systems, for all nodes over whole timeframe (230 s). (Diii) Mitochondrial encounter networks of these four simulated systems, at simulation times corresponding to experimentally determined networks in Figure 2E (singletons are omitted).

(E) Physical and network summary statistics are taken from the network of the final frame or overall from the time series (stated) for each system. Glossary: InterMitoMean, mean distance to the nearest neighboring mitochondrion (μm); MeanCH, mean convex hull area "swept out" by a trajectory ($\mu m^2$); network efficiency, average "closeness" (reciprocal of shortest path length) between pairs of nodes. See STAR methods (Summary statistics section) for full summary statistic definitions. See also Figure S5 and Table S1 (for full parameterizations).

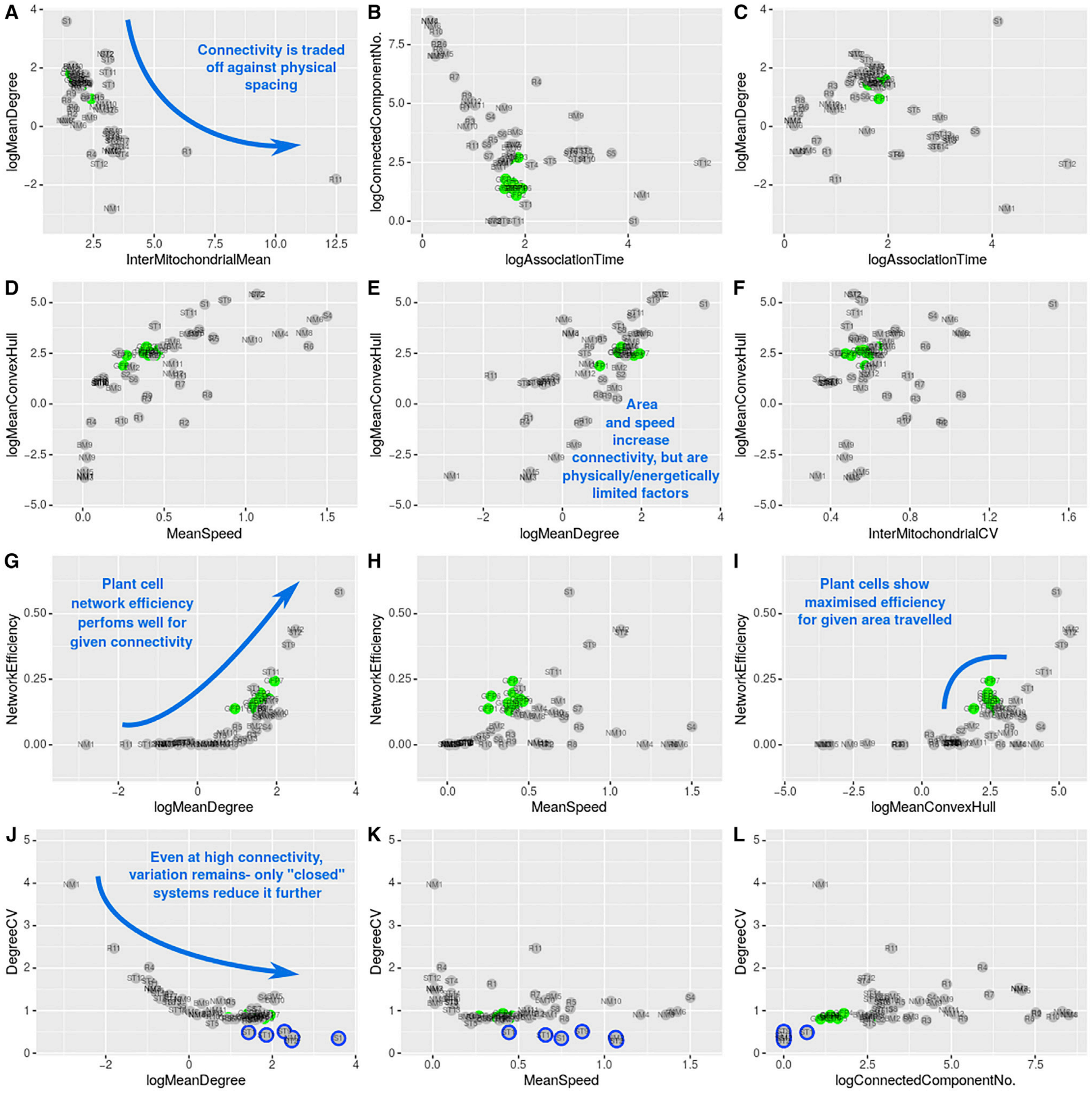


**Figure 5. Comparison of physical and social summary statistics between experimental and theoretical networks reveal tradeoffs and control principles of organelle dynamics**

Statistics plotted for experimental (green, n = 10) and simulated (gray, n = 54, different parameterizations, including the n = 4 (NM9-12) systems shown in Figure 4) networks over 230 seconds. Labels of individual datapoints reflect individual experiments (GFP*n*, n = 1–10, from single cell hypocotyl videos) and simulations (for example, random parameterizations as R; described fully in STAR methods and Table S1). Summaries of the relationships are as follows:

(A) Connectivity is traded off against physical spacing.
(B) Longer inter-mitochondrial association times theoretically increase component connectivity.
(C) Moderate association times encourage higher levels of connectivity in plant cells.
(D) Larger areas are traversed with increased speed, but physical limitations apply to plant cells.
(E) Larger areas covered increase network connectivity.
(F) Heterogeneity in physical spacing cannot be reduced without limiting area traversed and connectivity (as in E).
(G) Network efficiency is increased with connectivity and is almost maximal for plant cells.
(H and I) For a given speed and area, plant mitochondria mirror or exceed theoretically predicted network efficiency.
(J) Less connected networks have noisier connectivity values. An unavoidable level of variation is present across all networks—“closed” systems, without agent loss at cell edges, decrease this further.

*(legend continued on next page)*

a network, the more noise in its connectivity. However, this noise never decreases to zero – even highly connected networks that emerge from theoretical cellular dynamics have high CV values (0.5–1.5, Figure 5J). Increased mitochondrial speed, even exceeding biological values, cannot completely overcome this inevitable variability, even when the interaction network approaches a single connected component (Figures 5K and 5L).

Some parameterizations of our theoretical model suggest that a modest reduction in social heterogeneity may be possible without sacrificing the resolution to the spread-interaction tradeoff (blue circles, Figures 5J and 5K). However, the notable feature of this set of theoretical models is that they reflect "closed" systems, where mitochondria do not enter or leave the restricted 2D space under consideration. Model mitochondria in these simulations also cover consistently large areas and are never stationary. While this does not strictly reflect the behavior from our experimental observations (where organelles can enter and leave through the boundaries of the region), it does suggest another potential mechanism for resolving the spread-encounter tradeoff, namely by restricting the space in which organelles can travel.

Taken together, these results show that the mitochondrial motion adopted by plant cells both facilitates the spread of organelles throughout the cell and allows social encounters between these organelles. This motion is limited by physical constraints, and physical and social heterogeneity emerge as a necessary consequence of achieving high social connectivity under these constraints. Comparison of experimental and theoretical results suggests that the physical connectivity achieved by controlled mitochondrial dynamics in plant cells, given these constraints, provides a near-optimal foundation for the potential transfer of information across the chondriome.

### Genetic perturbation to mitochondrial dynamics transiently shifts the resolution to the physical-social tradeoff

To explore how the mitochondrial system addresses our hypothesized tradeoff when challenged, we turned to the *friendly* mutant, where the FRIENDLY protein, a determinant of mitochondrial dynamics, is disrupted. *Friendly* mitochondria are organized into large clusters of discrete organelles (Figure 6A), with an increase in organelle-level stress and several whole-plant phenotypes including shorter roots with more dead cells, reduced photosynthetic performance, lower biomass, and shorter etiolated hypocotyls (El Zawily et al., 2014). We hypothesized that the clustering of mitochondria in *friendly* would compromise the even spacing of organelles through the cell but might improve social connectivity, at least locally. Such a change would correspond to a "shift" on the tradeoff between physical spread and social connectivity: decreasing the former while increasing the latter.

Using a mutant line with both compromised FRIENDLY and mitochondrially localized GFP, again kindly provided by Prof. David Logan, we applied our above approach to characterize the physical and social behavior of mitochondria in *friendly*. We first observed that this shift in the physical-social tradeoff was apparent at short times. When observations were limited to a short time window, compared with the undisrupted wildtype, *friendly* mitochondria were clustered more closely, and their encounter networks had generally higher degree distributions (Figure 6B). However, and surprisingly, the *friendly* and wildtype behavior converged when longer time windows were considered. Over long time periods, the whole-cell statistics for inter-organelle spread and social degree grew more similar (Figure 6B). This suggests that *friendly* does not completely compromise the cell's ability to reach an appropriate resolution to the physical-social tradeoff but does challenge this resolution over short timescales. This transient challenge may explain why the *friendly* phenotypes are clear but not fatal.

How do *friendly* mitochondria manage to approach the long-term behavior of the wildtype? Further analysis of the physical and network statistics of *friendly* (Figures 6C–6E and S3) show that while speed and convex hull coverage are lower in *friendly*, as expected from their more limited motion, network efficiency and connected component structure are comparable with that in wildtype. This suggests that, although many mitochondria are physically constrained in clusters, a combination of cluster movement (El Zawily et al., 2014) and the motion of individual itinerant mitochondria between clusters helps to maintain the global network structure (Figure 6F). Rather than several, tightly internally connected but disparate cliques, this motion provides cross-clique connections and connects the global network. However, cluster motion is slow and individual highly itinerant mitochondria are rarer in *friendly* than in the wildtype. This relative scarcity of global connections is responsible for the slower timescale of *friendly* resolution to this tradeoff.

## DISCUSSION

We have combined experimental characterization and mechanistic modeling to show that plant mitochondria face a tension between "social" connectivity and physical spread in the cell. Strikingly, plant cells have evolved a resolution to this tension that produces social networks with high potential efficiency for information exchange. While other models have exploited network theory to represent mitochondrial connectivity (Sukhorukov *et al.*, 2012; Rafelski, 2013; Zamponi *et al.*, 2018; Picard and Sandi, 2021), we take a different, but complementary, perspective tailored to the specific nature of plant mitochondria. When mitochondria form large networks, it is typical to represent mitochondrial strands as edges and branch points as nodes, placing strong physical constraints on network structure (for example,

(K) Degree variation is fairly consistent across speed ranges.

(L) "Closed" systems demonstrate high connectivity, with low variation. Plant cells retain a necessary level of variation for similar connectivity. Blue circles denote theoretical simulations highlighted in the text (J, K, and L). Glossary: degree, number of direct neighbors of each node; InterMitoMean, mean distance to nearest neighboring mitochondrion (μm); MeanCH, mean convex hull area "swept out" by a trajectory ($\mu m^2$); network efficiency; average "closeness" (reciprocal of shortest path length) between pairs of nodes; connected components, number of connected subgraphs of the network; Association time, seconds spent in proximity to another agent/mitochondrion. Speed is in $\mu m\ s^{-1}$. See STAR methods (Summary statistics section) for full summary statistic definitions. See also Figures S2–S4 and S6 and Tables S1 and S2.

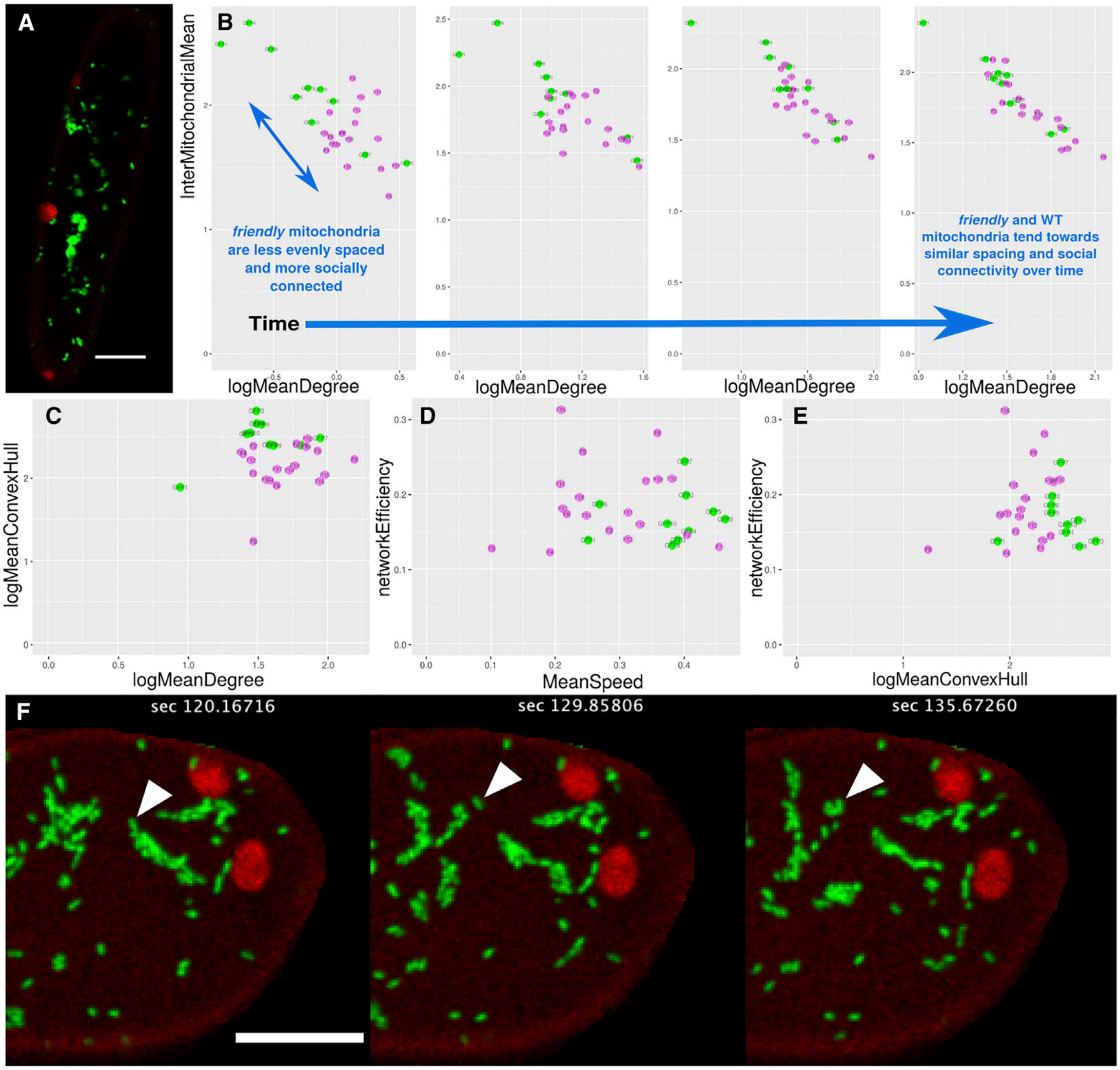


**Figure 6. Physical and social statistics of mitochondria in the *friendly* mutant compared with the wildtype**

(A) Example of mitochondria (green) in the *friendly* mutant, showing characteristic clustering, scale bar, 10 μm.

(B) Mean degree versus mean inter-mitochondrion distance in *friendly* (violet, n = 19) and wildtype (green, n = 10), calculated over different time windows (left to right—2, 39, 97, and 193 s). Initially, *friendly* shows higher degrees and lower physical spacing, with the difference decreasing over time. Note axes differ between plots.

(C–E) Further physical and social statistics of *friendly* compared with wildtype (from Figure 5).

(F) Itinerant mitochondria move between clusters in *friendly*, contributing to global connectivity. Highlighted individual mitochondrion (arrow) moves between clusters of mitochondria. Scale bar, 10 μm. Glossary: degree, number of direct neighbors of each node; InterMitoMean, mean distance to nearest neighboring mitochondrion (μm); MeanCH; mean convex hull area "swept out" by a trajectory ($\mu m^2$); network efficiency, average "closeness" (reciprocal of shortest path length) between pairs of nodes. Speed is in $\mu m\ s^{-1}$. See STAR methods (Summary statistics section) for full summary statistic definitions. See also Figure S3.

four-way branching is very rare). In plants, mitochondria are usually fragmented, individual organelles that interact through physical colocalization, so we represent organelles as nodes and colocalization events as edges. This picture is more analogous to the representation of social networks in other systems (including humans) where individuals interact, and physical constraints do not directly shape the network. Within this picture, we have used physical and network summary statistics to quantify spread, connectivity, association, and the potential for information transfer, and to demonstrate the cellular priorities of model plants and the energetic organelles within them.

We speculate that the balance of priorities between even spread and transient proximity may be a general principle underlying mitochondrial organization. In other plant cell types and over time, cell geometry varies substantially (Bassel and Smith, 2016); this hypothesis would predict that, given appropriate imaging technology in these cells, similar physical behavior and consequent network structures would be observed. In other organisms,

reticulated mitochondrial networks are more common (Hoitzing et al., 2015). An open question is whether the structure of these networks constitute another resolution to this tradeoff—achieving through network structure what plant organelles seem to achieve through dynamics. Both the network analysis and the flexible model parametrizations can readily be extended to whole-cell, three-dimensional mitochondrial dynamics, as well as to other organelles and cells of different geometries. To facilitate transferability to other systems, our code and data are publicly available (see resource availability). Ongoing developments in imaging technology will also enable further and tighter characterization of these rich, complex systems within the cell (Valm et al., 2017).

The work presented here sheds quantitative light on an open question: why the plant cell invests energy in driving the motion of the strikingly dynamic chondriome. Our results suggest that this motion helps resolve a tradeoff between physical spacing (which could be achieved by static organelles) and the capacity for communication and exchange (which cannot be achieved by static, spaced organelles). Several studies have discussed how beneficial exchange of genetic and protein material between organelles are beneficial for the cell (Arrieta-Montiel et al., 2009; Mouli et al., 2009; Xu et al., 2011; Patel et al., 2013; El Zawily et al., 2014). Here, we have shown that this is not without cost—to achieve this exchange, the cell must compromise even physical spread and induce heterogeneity. The specific influences of these facilitative physical dynamics with the protein and (sparse) genetic complements of plant mitochondria (Preuten *et al.*, 2010) is an exciting target for future research—how do physical mitochondrial dynamics shape genetic mtDNA populations (Johnston, 2019)? Characterization of encounter networks of mutant lines, where the machinery driving mitochondrial motion (El Zawily et al., 2014), or proteins regulating recombination of mtDNA (Arrieta-Montiel et al., 2009; Xu et al., 2011) are perturbed, will support and help develop this theory.

As with any complex biological system, it is likely that a combination of mechanistic influences gives rise to observed behavior. Accordingly, we do not intend to claim that our hypothesized tradeoff is the only mechanistic feature that shapes mitochondrial dynamics in plant cells. Colocalization with other organelles, including the endoplasmic reticulum, peroxisomes, plastids, and the nucleus, is likely important to facilitate metabolite exchange (Raghavendra and Padmasree, 2003; Scott et al., 2007; Rowland and Voeltz, 2012; Mueller and Reski, 2015; Shai et al., 2016; Van Dingenen et al., 2016; Barton et al., 2018). We have shown that the mode of motility can influence the behavior of the system, with a mix of diffusive motion and active cytoskeletal strand use increasing the connectivity and efficiency of the resulting networks of encounters. We have considered the spread of mitochondria throughout the cell as facilitating these encounters where required, but further specific studies will elucidate the finer-grained encounters prioritized under different circumstances. Further, ATP is required for motor protein motion, and local and cellular ATP levels (themselves shaped by mitochondrial distribution and function), thus, play a role in shaping mitochondrial motion. The development of detailed ATP reporters (De Col et al., 2017) will enable detailed characterization of this additional influence.

As a final concluding remark, plant bioenergetic behavior, and the role of mitochondria in shaping it, is recognized as a key target for further elucidation in the face of environmental change (Budar and Fujii, 2012). We hope that our work goes some way toward illustrating that these interdisciplinary approaches, quantitatively combining experimental characterization with mechanistic modeling, can help contribute to this important goal.

## STAR★METHODS

Detailed methods are provided in the online version of this paper and include the following:

- KEY RESOURCES TABLE
- RESOURCE AVAILABILITY
  - Lead contact
  - Materials availability
  - Data and code availability
- EXPERIMENTAL MODEL AND SUBJECT DETAILS
  - Plant growth
- METHOD DETAILS
  - Imaging
  - Video analysis
  - Independent video analysis
  - Network construction and analysis
  - Agent-based physical simulation
  - Theoretical models
  - Model behavior under different parameterisations
- QUANTIFICATION AND STATISTICAL ANALYSIS
  - Summary statistics
  - Additional summary statistics
  - Time dependency of summary statistics

## SUPPLEMENTAL INFORMATION

Supplemental information can be found online at https://doi.org/10.1016/j.cels.2021.04.006.

## ACKNOWLEDGMENTS

J.M.C. is supported by the BBSRC and University of Birmingham via the MIBTP doctoral training scheme (grant number BB/M01116X/1). This project has received funding from the European Research Council (ERC) under the European Union's Horizon 2020 research and innovation programme (grant agreement nos. 805046 (EvoConBiO) to I.G.J. and 715441 (GasPlaNt) to D.J.G.). G.W.B. acknowledges funding from BBSRC grant BB/S002804/1 and Leverhulme grants RPG-2016-049 and RPG-2019-267. We are very grateful to Prof. David Logan for mtGFP seeds and early discussions of the research plan. We gratefully acknowledge the Imaging Suite (BALM) at the University of Birmingham for support of imaging experiments and thank Alessandro di Maio and Prof. Markus Schwarzländer for advice with design and analysis of imaging experiments. We are also grateful to Konstantinos Giannakis and Arunas Radzvilavicius for valuable comments.

## AUTHOR CONTRIBUTIONS

I.G.J. conceived the project. J.M.C. performed experimental work and collected and analyzed image data. I.G.J. designed the model. I.G.J. and J.M.C. wrote the analysis code. I.G.J., J.M.C., and G.W.B. designed the experimental setup. J.M.C. and I.G.J. analyzed the model. I.G.J., G.W.B., and D.J.G. supervised the experimental work. J.M.C. and I.G.J. drafted the manuscript. All authors edited the manuscript and wrote the final version.

## DECLARATION OF INTERESTS

The authors declare no competing interests.

Received: December 4, 2020
Revised: February 22, 2021
Accepted: April 13, 2021
Published: May 5, 2021

## REFERENCES

Agrawal, A., Pekkurnaz, G., and Koslover, E.F. (2018). Spatial control of neuronal metabolism through glucose-mediated mitochondrial transport regulation. eLife *7*, e40986. https://doi.org/10.7554/eLife.40986.

Arimura, S. -i., Yamamoto, J., Aida, G.P., Nakazono, M., and Tsutsumi, N. (2004). Frequent fusion and fission of plant mitochondria with unequal nucleoid distribution. Proc. Natl. Acad. Sci. USA *101*, 7805–7808. https://doi.org/10.1073/pnas.0401077101.

Arimura, S.I. (2018). Fission and fusion of plant mitochondria, and genome maintenance. Plant Physiol *176*, 152–161. https://doi.org/10.1104/pp.17.01025.

Arrieta-Montiel, M.P., Shedge, V., Davila, J., Christensen, A.C., and Mackenzie, S.A. (2009). Diversity of the Arabidopsis mitochondrial genome occurs via nuclear-controlled recombination activity. Genetics *183*, 1261–1268. https://doi.org/10.1534/genetics.109.108514.

Avisar, D., Abu-Abied, M., Belausov, E., Sadot, E., Hawes, C., and Sparkes, I.A. (2009). A comparative study of the involvement of 17 Arabidopsis myosin family members on the motility of Golgi and other organelles. Plant Physiol *150*, 700–709.

Barton, K.A., Wozny, M.R., Mathur, N., Jaipargas, E.A., and Mathur, J. (2018). Chloroplast behaviour and interactions with other organelles in Arabidopsis thaliana pavement cells. J. Cell Sci. *131*, jcs202275. https://doi.org/10.1242/jcs.202275.

Bassel, G.W., and Smith, R.S. (2016). Quantifying morphogenesis in plants in 4D. Curr. Opin. Plant Biol. *29*, 87–94. https://doi.org/10.1016/j.pbi.2015.11.005.

Bauwe, H., Hagemann, M., and Fernie, A.R. (2010). Photorespiration: players, partners and origin. Trends Plant Sci *15*, 330–336. https://doi.org/10.1016/j.tplants.2010.03.006.

Breuer, D., Nowak, J., Ivakov, A., Somssich, M., Persson, S., and Nikoloski, Z. (2017). System-wide organization of actin cytoskeleton determines organelle transport in hypocotyl plant cells. Proc. Natl. Acad. Sci. USA *114*, E5741–E5749. https://doi.org/10.1073/pnas.1706711114.

Budar, F., and Fujii, S. (2012). Cytonuclear adaptation in plants. Adv. Bot. Res. *63*, 99–126. https://doi.org/10.1016/B978-0-12-394279-1.00005-3.

Chen, H., and Chan, D.C. (2006). Critical dependence of neurons on mitochondrial dynamics. Curr Opin Cell Biol *18*, 453–459. https://doi.org/10.1016/j.ceb.2006.06.004.

Cohen, S., Valm, A.M., and Lippincott-Schwartz, J. (2018). Interacting organelles. Curr. Opin. Cell Biol. *53*, 84–91. https://doi.org/10.1016/j.ceb.2018.06.003.

De Col, V., Fuchs, P., Nietzel, T., Elsässer, M., Voon, C.P., Candeo, A., Seeliger, I., Fricker, M.D., Grefen, C., Møller, I.M., et al. (2017). ATP sensing in living plant cells reveals tissue gradients and stress dynamics of energy physiology. eLife *6*, e26770, https://doi.org/10.7554/eLife.26770.

Doniwa, Y., Arimura, S., and Tsutsumi, N. (2007). Mitochondria use actin filaments as rails for fast translocation in Arabidopsis and tobacco cells. Plant Biotechnol *24*, 441–447.

Ekanayake, S.B., El Zawily, A.M., Paszkiewicz, G., Rolland, A., and Logan, D.C. (2015). Imaging and analysis of mitochondrial dynamics in living cells. Methods Mol. Biol. *1305*, 223–240.

El Zawily, A.M., Schwarzländer, M., Finkemeier, I., Johnston, I.G., Benamar, A., Cao, Y., Gissot, C., Meyer, A.J., Wilson, K., Datla, R., et al. (2014). FRIENDLY regulates mitochondrial distribution, fusion, and quality control in Arabidopsis. Plant Physiol *166*, 808–828.

Feng, X., Arimura, S., Hirano, H.Y., Sakamoto, W., and Tsutsumi, N. (2004). Isolation of mutants with aberrant mitochondrial morphology from Arabidopsis thaliana. Genes Genet. Syst. *79*, 301–305.

Figge, M.T., Reichert, A.S., Meyer-Hermann, M., and Osiewacz, H.D. (2012). Deceleration of fusion-fission cycles improves mitochondrial quality control during aging. PLoS Comput. Biol. *8*, e1002576.

Frederick, R.L., and Shaw, J.M. (2007). Moving mitochondria: establishing distribution of an essential organelle. Traffic *8*, 1668–1675.

Hoitzing, H., Johnston, I.G., and Jones, N.S. (2015). What is the function of mitochondrial networks? A theoretical assessment of hypotheses and proposal for future research. BioEssays *37*, 687–700.

Hyde, B.B., Twig, G., and Shirihai, O.S. (2010). Organellar vs cellular control of mitochondrial dynamics. Semin. Cell Dev. Biol. *21*, 575–581.

Islam, M.S., Niwa, Y., and Takagi, S. (2009). Light-dependent intracellular positioning of mitochondria in Arabidopsis thaliana mesophyll cells. Plant Cell Physiol *50*, 1032–1040.

Jacoby, R.P., Li, L., Huang, S., Pong Lee, C., Millar, A.H., and Taylor, N.L. (2012). Mitochondrial composition, function and stress response in plants. J. Integr. Plant Biol. *54*, 887–906.

Jaipargas, E.A., Barton, K.A., Mathur, N., and Mathur, J. (2015). Mitochondrial pleomorphy in plant cells is driven by contiguous ER dynamics. Front. Plant Sci. *6*, 783.

Johnston, I.G. (2019). Tension and resolution: dynamic, evolving populations of organelle genomes within plant cells. Mol. Plant *12*, 764–783.

Jones, D.P. (1986). Intracellular diffusion gradients of O2 and ATP. Am. J. Physiol. Cell Physiol. *250*, C663–C675.

Karbowski, M., and Youle, R.J. (2003). Dynamics of mitochondrial morphology in healthy cells and during apoptosis. Cell Death Differ *10*, 870–880.

Liesa, M., and Shirihai, O.S. (2013). Mitochondrial dynamics in the regulation of nutrient utilization and energy expenditure. Cell Metab *17*, 491–506.

Liu, X., Weaver, D., Shirihai, O., and Hajnóczky, G. (2009). Mitochondrial "kiss-and-run": interplay between mitochondrial motility and fusion–fission dynamics. EMBO J *28*, 3074–3089.

Logan, D.C. (2003). Mitochondrial dynamics. New Phytol *160*, 463–478.

Logan, D.C. (2006). Plant mitochondrial dynamics. Biochim. Biophys. Acta *1763*, 430–441.

Logan, D.C. (2007). The mitochondrial compartment. J. Exp. Bot. *58*, 1225–1243.

Logan, D.C. (2010a). Mitochondrial fusion, division and positioning in plants. Biochem. Soc. Trans. *38*, 789–795.

Logan, D.C. (2010b). The dynamic plant chondriome. Semin. Cell Dev. Biol. *21*, 550–557.

Logan, D.C., and Leaver, C.J. (2000). Mitochondria-targeted GFP highlights the heterogeneity of mitochondrial shape, size and movement within living plant cells. J. Exp. Bot. *51*, 865–871.

Mano, S., Miwa, T., Nishikawa, S., Mimura, T., and Nishimura, M. (2008). The plant organelles database (PODB): a collection of visualized plant organelles and protocols for plant organelle research. Nucleic Acids Res *36*, D929–D937.

Mano, S., Miwa, T., Nishikawa, S., Mimura, T., and Nishimura, M. (2009). Seeing is Believing: on the use of image databases for visually exploring plant organelle dynamics. Plant Cell Physiol *50*, 2000–2014.

Mano, S., Nakamura, T., Kondo, M., Miwa, T., Nishikawa, S., Mimura, T., Nagatani, A., and Nishimura, M. (2014). The plant organelles database 3 (PODB3) update 2014: integrating electron micrographs and new options for plant organelle research. Plant Cell Physiol *55*, e1.

Mano, S., Miwa, T., Nishikawa, S.-I., Mimura, T., and Nishimura, M. (2011). The plant organelles database 2 (PODB2): an updated resource containing movie data of plant organelle dynamics. Plant Cell Physiol *52*, 244–253.

Mouli, P.K., Twig, G., and Shirihai, O.S. (2009). Frequency and selectivity of mitochondrial fusion are key to its quality maintenance function. Biophys. J. *96*, 3509–3518.

Mueller, S.J., and Reski, R. (2015). Mitochondrial dynamics and the ER: the plant perspective. Front Cell Dev Biol *3*, 78.

Okamoto, K., and Shaw, J.M. (2005). Mitochondrial morphology and dynamics in yeast and multicellular eukaryotes. Annu. Rev. Genet. *39*, 503–536.

Patel, P.K., Shirihai, O., and Huang, K.C. (2013). Optimal dynamics for quality control in spatially distributed mitochondrial networks. PLoS Comput. Biol. *9*, e1003108.

Peremyslov, V.V., Cole, R.A., Fowler, J.E., and Dolja, V.V. (2015). Myosin-powered membrane compartment drives cytoplasmic streaming, cell expansion and plant development. PLOS one *10*, e0139331.

Picard, M., and Sandi, C. (2021). The social nature of mitochondria: implications for human health. Neurosci. Biobehav. Rev. *120*, 595–610.

Preuten, T., Cincu, E., Fuchs, J., Zoschke, R., Liere, K., and Börner, T. (2010). Fewer genes than organelles: extremely low and variable gene copy numbers in mitochondria of somatic plant cells. Plant J *64*, 948–959.

Rafelski, S.M. (2013). Mitochondrial network morphology: building an integrative, geometrical view. BMC Biol *11*, 71.

Raghavendra, A.S., and Padmasree, K. (2003). Beneficial interactions of mitochondrial metabolism with photosynthetic carbon assimilation. Trends Plant Sci *8*, 546–553.

Rowland, A.A., and Voeltz, G.K. (2012). Endoplasmic reticulum-mitochondria contacts: function of the junction. Nat. Rev. Mol. Cell Biol. *13*, 607–625.

Sage, R.F., Sage, T.L., and Kocacinar, F. (2012). Photorespiration and the evolution of C4 photosynthesis. Annu. Rev. Plant Biol. *63*, 19–47.

Sbalzarini, I.F., and Koumoutsakos, P. (2005). Feature point tracking and trajectory analysis for video imaging in cell biology. J. Struct. Biol. *151*, 182–195.

Schuler, M.H., Lewandowska, A., Caprio, G.D., Skillern, W., Upadhyayula, S., Kirchhausen, T., Shaw, J.M., and Cunniff, B. (2017). Miro1-mediated mitochondrial positioning shapes intracellular energy gradients required for cell migration. Mol. Biol. Cell *28*, 2159–2169.

Scott, I., and Logan, D.C. (2008). Mitochondrial morphology transition is an early indicator of subsequent cell death in Arabidopsis. New Phytol *177*, 90–101.

Scott, I., Sparkes, I.A., and Logan, D.C. (2007). The missing link: inter-organellar connections in mitochondria and peroxisomes? Trends Plant Sci *12*, 380–381.

Seguí-Simarro, J.M., Coronado, M.J., and Staehelin, L.A. (2008). The mitochondrial cycle of Arabidopsis shoot apical meristem and leaf primordium meristematic cells is defined by a perinuclear tentaculate/cage-like mitochondrion. Plant Physiol *148*, 1380–1393.

Seguí-Simarro, J.M., and Staehelin, L.A. (2009). Mitochondrial reticulation in shoot apical meristem cells of Arabidopsis provides a mechanism for homogenization of mtDNA prior to gamete formation. Plant Signal. Behav. *4*, 168–171.

Shai, N., Schuldiner, M., and Zalckvar, E. (2016). No peroxisome is an island - Peroxisome contact sites. Biochim. Biophys. Acta *1863*, 1061–1069.

Shaw, J.M., and Nunnari, J. (2002). Mitochondrial dynamics and division in budding yeast. Trends Cell Biol *12*, 178–184.

Shimmen, T., and Yokota, E. (2004). Cytoplasmic streaming in plants. Curr. Opin. Cell Biol. *16*, 68–72.

Shutt, T.E., and McBride, H.M. (2013). Staying cool in difficult times: mitochondrial dynamics, quality control and the stress response. Biochim. Biophys. Acta *1833*, 417–424.

Spillane, M., Ketschek, A., Merianda, T.T., Twiss, J.L., and Gallo, G. (2013). Mitochondria coordinate sites of axon branching through localized intra-axonal protein synthesis. Cell Rep *5*, 1564–1575.

Sukhorukov, V.M., Dikov, D., Reichert, A.S., and Meyer-Hermann, M. (2012). Emergence of the mitochondrial reticulum from fission and fusion dynamics. PLoS Comput. Biol. *8*, e1002745.

Takanashi, H., Arimura, S., Sakamoto, W., and Tsutsumi, N. (2006). Different amounts of DNA in each mitochondrion in rice root. Genes Genet. Syst. *81*, 215–218.

Tinevez, J.Y., Perry, N., Schindelin, J., Hoopes, G.M., Reynolds, G.D., Laplantine, E., Bednarek, S.Y., Shorte, S.L., and Eliceiri, K.W. (2017). TrackMate: An open and extensible platform for single-particle tracking. Methods *115*, 80–90.

Tominaga, M., and Ito, K. (2015). The molecular mechanism and physiological role of cytoplasmic streaming. Curr. Opin. Plant Biol. *27*, 104–110.

Twig, G., Elorza, A., Molina, A.J., Mohamed, H., Wikstrom, J.D., Walzer, G., Stiles, L., Haigh, S.E., Katz, S., Las, G., et al. (2008). Fission and selective fusion govern mitochondrial segregation and elimination by autophagy. EMBO J *27*, 433–446.

Valm, A.M., Cohen, S., Legant, W.R., Melunis, J., Hershberg, U., Wait, E., Cohen, A.R., Davidson, M.W., Betzig, E., and Lippincott-Schwartz, J. (2017). Applying systems-level spectral imaging and analysis to reveal the organelle interactome. Nature *546*, 162.

Van Dingenen, J., Blomme, J., Gonzalez, N., and Inzé, D. (2016). Plants grow with a little help from their organelle friends. J. Exp. Bot. *67*, 6267–6281. https://doi.org/10.1093/jxb/erw399.

Van Gestel, K., Köhler, R.H., and Verbelen, J.P. (2002). Plant mitochondria move on F-actin, but their positioning in the cortical cytoplasm depends on both F-actin and microtubules. J. Exp. Bot. *53*, 659–667.

Viana, M.P., Brown, A.I., Mueller, I.A., Goul, C., Koslover, E.F., and Rafelski, S.M. (2020). Mitochondrial fission and fusion dynamics generate efficient, robust, and evenly distributed network topologies in budding yeast cells. Cell Syst *10*, 287–297.e5.

Wang, P., and Hussey, P.J. (2015). Interactions between plant endomembrane systems and the actin cytoskeleton. Front. Plant Sci. *6*, 422.

Wang, Y., Berkowitz, O., Selinski, J., Xu, Y., Hartmann, A., and Whelan, J. (2018). Stress responsive mitochondrial proteins in Arabidopsis thaliana. Free Radic. Biol. Med. *122*, 28–39.

Welchen, E., García, L., Mansilla, N., and Gonzalez, D.H. (2014). Coordination of plant mitochondrial biogenesis: keeping pace with cellular requirements. Front. Plant Sci. *4*, 551.

Whelan, J., and Murcha, M.W. (2015). Plant Mitochondria: Methods and Protocols, Plant mitochondria: Methods and Protocols (Springer).

Williams, C.F., and George, C.H. (2019). Connect and conquer: collectivized behavior of mitochondria and bacteria. Front Physiol *10*, 340.

Xu, Y.Z., Arrieta-Montiel, M.P., Virdi, K.S., de Paula, W.B., Widhalm, J.R., Basset, G.J., Davila, J.I., Elthon, T.E., Elowsky, C.G., Sato, S.J., et al. (2011). MutS HOMOLOG1 is a nucleoid protein that alters mitochondrial and plastid properties and plant response to high light. Plant Cell *23*, 3428–3441.

Yoshinaga, K., Arimura, S., Niwa, Y., Tsutsumi, N., Uchimiya, H., and Kawai-Yamada, M. (2005). Mitochondrial behaviour in the early stages of ROS stress leading to cell death in Arabidopsis thaliana. Ann. Bot. *96*, 337–342.

Yu, Y., Lee, H.C., Chen, K.C., Suhan, J., Qiu, M., Ba, Q., and Yang, G. (2016). Inner membrane fusion mediates spatial distribution of axonal mitochondria. Sci. Rep. *6*, 18981.

Zamponi, N., Zamponi, E., Cannas, S.A., Billoni, O.V., Helguera, P.R., and Chialvo, D.R. (2018). Mitochondrial network complexity emerges from fission/fusion dynamics. Sci. Rep. *8*, 363.

Zheng, M., Beck, M., Müller, J., Chen, T., Wang, X., Wang, F., Wang, Q., Wang, Y., Baluska, F., Logan, D.C., et al. (2009). Actin turnover is required for myosin-dependent mitochondrial movements in arabidopsis root hairs. PLoS one *4*, e5961.

Zorov, D.B., Juhaszova, M., and Sollott, S.J. (2014). Mitochondrial reactive oxygen species (ROS) and ROS-induced ROS release. Physiol. Rev. *94*, 909–950.

## STAR★METHODS

### KEY RESOURCES TABLE

| REAGENT or RESOURCE | SOURCE | IDENTIFIER |
|---|---|---|
| Chemicals, peptides, and recombinant proteins | | |
| Propidium Iodide solution (1mg/mL in water) | Sigma-Aldrich | P4864; CAS: 25535-16-4 |
| Murashige and Skoog Basal Medium | Sigma-Aldrich | M5519 |
| Agar Powder | Fisher Scientific | CAS: 9002-18-0 |
| Deposited data | | |
| Mitochondrial-targeted GFP Wild-type single cell confocal videos | This paper | https://github.com/StochasticBiology/plant-mitochondria/tree/master/Videos_AthalianaGFP |
| Experimental models: organisms/strains | | |
| Mitochondrial-targeted GFP *A. thaliana* | Prof. David Logan, Logan and Leaver, 2000 | N/A |
| Mitochondrial-targeted GFP *friendly A. thaliana* | Prof. David Logan, El Zawily et al., 2014 | N/A |
| Software and algorithms | | |
| ZEN Digital Imaging for Light Microscopy | Zeiss | RRID:SCR_013672 |
| ImageJ 2.0.0 (within Fiji) | | RRID:SCR_003070/ RRID:SCR_002285 |
| TrackMate ImageJ plugin | Tinevez et al., 2017 | N/A |
| Mosaic ParticleTracker Plugin | National Center for Microscopy and Imaging Research, Sbalzarini and Koumoutsakos, 2005 | RRID:SCR_001935 |
| Mathematica 11.3.0.0 | Wolfram Mathematica | RRID:SCR_014448 |
| R version 4.0.1 | The R Foundation for Statistical Computing | https://www.r-project.org/ |
| Other | | |
| Zeiss LSM 710 Confocal Inverted Microscope | Zeiss | RRID:SCR_018063 |
| Zeiss LSM 900 with Airyscan 2 | Zeiss | N/A |

### RESOURCE AVAILABILITY

#### Lead contact

Further information and requests for resources and reagents should be directed to and will be fulfilled by the Lead Contact, Iain G. Johnston (Iain.Johnston@uib.no).

#### Materials availability

This study did not generate new materials.

#### Data and code availability

- Acquired videos of mitochondrial dynamics have been deposited at GitHub and are publicly available at https://github.com/StochasticBiology/plant-mitochondria
- Original code is also publicly available at the link above.
- The scripts used to generate the figures reported in this paper are available at the link above.
- Any additional information required to reproduce this work is available from the Lead Contact.

### EXPERIMENTAL MODEL AND SUBJECT DETAILS

#### Plant growth

Seeds of *Arabidopsis thaliana* with mitochondrial-targeted GFP (kindly provided by Prof. David Logan (Logan and Leaver, 2000; El Zawily et al., 2014)) were surface sterilized in 50% (v/v) household bleach solution for 4 minutes with continual inversion, rinsed three times with sterile water, and plated onto ½ MS Agar. Plated seeds were stratified in the dark for 2 days at 4°C. Seedlings were grown in 16hr light/8hr dark at 21°C for 4-5 days before use.

## METHOD DETAILS

### Imaging

Prior to mounting, cell walls were stained with 10μM Propidium Iodide (PI) solution for 3 minutes. Following a protocol modified from (Whelan and Murcha, 2015), full seedlings were mounted in water on microscope slides, with cover slip. Imaging of dynamic systems in living cells is a balance between spatial/temporal resolution and maintaining physiological conditions. To avoid undesirable perturbations to the system including physical and light stress and hypoxia, all imaging was done maintaining low laser intensities and within at most 10 minutes of mounting to minimise the effects of physical stress and hypoxia (Prof Markus Schwarzländer, personal communication).

A Zeiss 710 laser scanning confocal microscope was used to capture time lapse images. To test robustness of the imaging protocol, a Zeiss 900 with AiryScan 2 detector was also used for several identically prepared samples, with no differences between summary statistics collected from these samples and those from the 710 beyond natural variability. For cellular characterisation we used excitation wavelength 543nm, detection range 578-718nm for both chlorophyll autofluorescence (peak emission 679.5nm) and for PI (peak emission 648nm). For mitochondrial capture we used excitation wavelength 488nm, detection range 494-578nm for GFP (peak emission 535.5nm).

### Video analysis

Individual cells were cropped from the acquired video data using the cell wall PI signal using ImageJ. The size of each video was scaled to the universal length scale 5.0 pixels/μm. We then extracted individual mitochondrial trajectories from the acquired video data using TrackMate (Tinevez et al., 2017) in ImageJ 2.0.0. Typical settings used were application of the LoG Detector filter with a blob diameter of 1μm and threshold of 2-7, filters were set on spot quality if deemed necessary. The Simple LAP Tracker was run with a linking max distance of 4μm, gap-closing distance of 5μm and gap-closing max frame gap of 2 frames. In each case we visually confirmed that individual mitochondria were appropriately highlighted and that tracks were well captured, editing occasional tracks where necessary. Alternative tracking software was used to check the robustness of this approach (Mosaic Particle-Tracker (Sbalzarini and Koumoutsakos, 2005))

### Independent video analysis

Independent videos were analysed using the same video and encounter network analysis described in STAR methods. Independent video 1 showed Kaede fluorescent mitochondria in the cotyledon of *Arabidopsis thaliana* taken from (Arimura et al., 2004) via PODB3 (Mano et al., 2008, 2009, 2011, 2014). Independent video 2 showed TMRM stained mitochondria in the hypocotyl of *Arabidopsis thaliana* taken from (El Zawily et al., 2014).

### Network construction and analysis

We represent physical colocalisations of mitochondria as an undirected network, where a node is a mitochondrion and an edge between two nodes reflects historical colocalisation. Colocalisation was defined as being separated by at most 1.6μm (the characteristic length scale of a mitochondrion), and is not necessarily a fusion event. Custom code in Mathematica 11.3.0.0 was used to compute network statistics.

### Agent-based physical simulation

Our mechanistic simulation models $n_{mito}$ individual mitochondrial agents in a rectangular simulation cell (30 x 100 μm, based on average micron lengths/widths of sampled cells). $N_{cyt}$ linear cytoskeletal strands are randomly positioned in the cell. Mitochondrial agents within a distance $d_{cyt}$ of a strand can attach and detach to and from that strand according to Poisson processes with rates $k_{on}$ and $k_{off}$ respectively. Mitochondrial agents move diffusively with diffusion coefficient D when not attached to a strand; when attached they move with speed $k_{cyt}$. Agents can switch strands at cytoskeletal intersections. When mitochondrial agents are within a distance $d_{mito}$ of another agent, their diffusion coefficient is scaled by parameter $k_{mito}$, reflecting possible slowing on colocalisation. When within a boundary region of the cell (width 4 μm), agents have a rate $k_{out}$ of disappearance (modelling movement out of the cytosol plane); when an agent disappears, a new agent is created at a random point within the boundary region, in order that $n_{mito}$ is constant. Physical steps that would remove agents from the cell are truncated (the edge removal process randomly removes agents from the cell periphery and replaces them at a random position in the periphery). Nearby agents influence each other with hydrodynamic force $F_{hydro}$ scaled by distance to a partner, reflecting cytoplasmic streaming. The simple model of hydrodynamic influence (not intended to solve real fluid equations of motion) comprises an averaging of neighbouring velocities scaled by the reciprocal distance to a neighbour. The simulation is run for an initialisation period of $10^3$ frames before data capture, determined with preliminary investigation to be sufficient to remove influence of initial conditions. The model is summarised in Figure 3.

### Theoretical models

Theoretical models were used in the network analysis, and covered a wide range of behaviors. All parameterisations for the 54 simulations used can be seen in Table S1. All NM and ST models had *N*=100 mitochondria. Values were chosen to highlight the influence of different parameterisations on the simulation.

Those labelled NM were the simplest models, with changes only occurring in the diffusion rate (D), hydrodynamic force ($F_{hydro}$) and edge disappearance rate ($k_{out}$), with the exception of NM11 where cytoskeletal strand use is introduced.

BM models all used cytoskeletal strands, and had $n_{mito}$=198 agents. The differences were in their diffusion rate (BM1-4,7,9 had values between D=0 $\mu m^2/s$ and D=0.85$\mu m^2/s$) and hydrodynamic forces (BM5-8,10 had D=0.52 $\mu m^2/s$ and a range from $F_{hydro}$=0 to $F_{hydro}$=0.8).

ST 3-8,10,13,14 were simple models without strands, with D=0.086 $\mu m^2/s$. The remaining ST models were simple models without cytoskeletal strands with D values between 0 and 1.

R1-11 are a collection of models generated with completely random parameters between the values set as priors, with for example, $n_{mito}$ between 9 and 191, $n_{cyt}$ between 0 and 13, and *D* between 0.152 and 0.705 $\mu m^2/s$.

S models were chosen deliberately due to their behaviors reflecting informative limits of possible dynamics, with S1 having agents clustering at polar ends of the cell, due to high $k_{cyt}$ and $k_{out}$ = 0 $s^{-1}$. S2 formed networks with very 'strand-like' formations, with many agents around the outside of the cell appearing and disappearing. S4 had many cytoskeletal strands, high $k_{on}$ and low $k_{off}$. This led to a very strong streaming effect, with agents moving constantly from one end of the cell to the other in a continuous wave. S5 agents barely moved, with a low diffusion rate and no strand use. S6 had an intermediate diffusion rate, and no strand use, but still formed into a well-connected network. S7 had a very high diffusion rate and no stands, leading to a system in which agents moved about very quickly, and formed into a very dense network.

### Model behavior under different parameterisations

Further exploration of different parameterisations of the model are illustrated in Figure S5, where we vary the rate of agent loss at the boundary region ($k_{out}$, Figures S5A and S5B), a parameter that is independent of motion and transport rates of agents in the system. $k_{out}$ mirrors motion out of the cytosolic plane during imaging of plant mitochondria. Results are robust for a range of low $k_{out}$ values (Figures 4 and S5A), and it is only when reaching high probabilities of loss from the edge that networks structure substantially varies (Figure S5B). We chose values for $k_{out}$ based on initial empirical observations. Further to this comparison, we also alter the ratio of $k_{on}$ to $k_{off}$ (Figures S5C–S5F). Results demonstrate that it is the ratio of $k_{on}$ to $k_{off}$ that influences agent motion and network structure (Figures S5C–S5H). Higher $k_{on}$ : $k_{off}$ consistently gives an increase in connectivity, area traveled, and network efficiency (Table 2 C-H). We also compare changes in hydrodynamic force ($F_{hydro}$, Figures S5I and S5J), speed of agents on strands ($k_{cyt}$, Figures S5K and S5L), motion scaling of interacting mitochondria ($k_{mito}$, Figures S5M and S5N), the interaction range for mitochondria motion scaling, with $k_{mito}$=0 ($d_{mito}$, Figures S5O and S5P), and $k_{mito}$ incremental increase with a constant $d_{mito}$ (Q-S). Summary statistics and specific parameterisations for each model can be seen in Table S2. In each case, as with Figures 4 and 5, these models are generated to describe the range of behaviors of the system, not an explicit inference of exact values.

## QUANTIFICATION AND STATISTICAL ANALYSIS

### Summary statistics

Analysis of mitochondrial motion used both physical and network statistics. Physical statistics included speed ($\mu ms^{-1}$), taken as distance moved per frame per track, averaged over all mitochondria in all frames. Diffusion rate was estimated from the slope of mean-square-displacement (MSD) over time, taken over *n*=9 mitochondria observed to be moving diffusively (not moving in a linear fashion, and covering small areas).

Angle of motion was calculated in degrees as

$$180 - \left(\frac{180}{\pi}\cos^{-1}\frac{\boldsymbol{a}\cdot\boldsymbol{b}}{|\boldsymbol{a}||\boldsymbol{b}|}\right)$$

where ***a*** and ***b*** were vectors between three coordinates over three frames. Angle distributions represent all mitochondria in all frames. Convex hull area was calculated as the area ($\mu m^2$) within a polygon traced around all furthest reaching points in a trajectory of frame length $\geq$3. Convex hull values represent all mitochondria in all frames, unless otherwise stated. Minimum inter-mitochondrial distance ($\mu$m) was the minimum Euclidean distance between each mitochondrion and its nearest neighbour in each frame, unless otherwise stated. Association time was taken as the length, in seconds, for which two individual mitochondria were paired in the adjacency matrices, taken over all mitochondria and all frames, unless otherwise stated.

Networks of encounters were built from adjacency matrices when individual mitochondria colocalised. Networks were historical, building on colocalisations from previous frames. For network statistics based on connectivity, singleton counts were also included, representing mitochondria that were present in the system at a time frame, but did not colocalise with others.

All following network statistics were taken for networks at the final frame of videos, unless otherwise stated. Connected component number is the number of subgraphs in which nodes are internally connected, and not externally connected to any other subgraphs. Degree values give the immediate number of neighbours of a node. Network efficiency is the sum of the shortest distance between all nodes in the network, averaged over the number of nodes, including singletons. Network efficiency was calculated as

$$E(G) = \frac{1}{n(n-1)}\sum_{i\neq j\in G}\frac{1}{d(i,j)}$$

where *G* is the network of interest, *n* is the number of nodes in the network and *d(i, j)* is the distance (edge number) between node *i* and node *j*. *E(G)* for weighted graphs (used only in Figure S6) is calculated using the sum of 1/association time.

Number of individual cells and/or models used in each analysis are provided in figure legends.

### Additional summary statistics

As well as summary statistics included in the main text, Figure S3 also includes extra summary statistics. All following network statistics were taken for networks at the final frame of videos, unless otherwise stated. The average number of connected neighbours is the number of other nodes each node within a connected component can reach, over all nodes- including singletons (ie the social circle of mitochondria). Percolation threshold was taken as the frame number at which the largest connected component showed a 50% increase in size from the previous frame. Degree drop is the difference in degree value between highest degree node, and a node 5 random node-by-node steps away (random walks). This value is taken for 200 random walks, normalised by the number of nodes in the network, and averaged.

### Time dependency of summary statistics

As these dynamic networks evolve over time, with more encounters being added as the history of the population builds up, the values of several network summary statistics depend on sampling time. Physical statistics do not depend on history and therefore remain consistent across sampling times, including mean speed (Figures 5D and S2iv), and inter-mitochondrion distance (Figures 5A and S2i). Some statistics predictably change, such as an increase in the area travelled at later time points (Figures 5E and S2v). One consistent feature across plant cell networks is the decrease in connected component number over time as networks condense and build up (Figures 5B and S2ii), also shown by the increase in connectivity over time, shown by mean degree (Figures 5J and S2x). There is also a decrease in variability over time within the networks, as shown by degree variation, due to the averaging and dampening or extremes in degree values as networks become more connected (Figures 5J, 5L, and S2x–S2xii). Although not drastic, changes in network statistics over time reveal the nature of the system as it is captured, with the preservation of physical statistics such as speed and spread of mitochondria across the cell.

Cell Systems, Volume *12*

## Supplemental information

# Network analysis of *Arabidopsis* mitochondrial dynamics reveals a resolved tradeoff between physical distribution and social connectivity

**Joanna M. Chustecki, Daniel J. Gibbs, George W. Bassel, and Iain G. Johnston**

***Network analysis of Arabidopsis mitochondrial dynamics reveals a resolved tradeoff between physical distribution and social connectivity***

**Combined Supplementary Information PDF**

Figure S1: Networks and summary statistics from two independent videos of mitochondrial motion, related to Figures 2 and 5.

Figure S2. Physical and social statistics at different timepoints, related to Figure 5.

Figure S3. All comparisons of physical and social statistics analysed, related to Figures 5 and 6.

Figure S4. Comparison of physical and social summary statistics between experimental and theoretical networks, enforcing adjacency for encounters between mitochondria, related to Figure 5.

Figure S5. Exploration of a range of simulation parameterisations showing the range of possible behaviours of agents in the cell and networks of encounters, related to Figure 4.

Figure S6. Network efficiency of unweighted and weighted interaction networks, related to Figure 5.

Supplementary Table 1. Simulation parameterisations, related to Figures 4,5, and S2-4.

Supplementary Table 2. Simulation parameterisations for parameter sweeps, related to Figures 5 and S5.

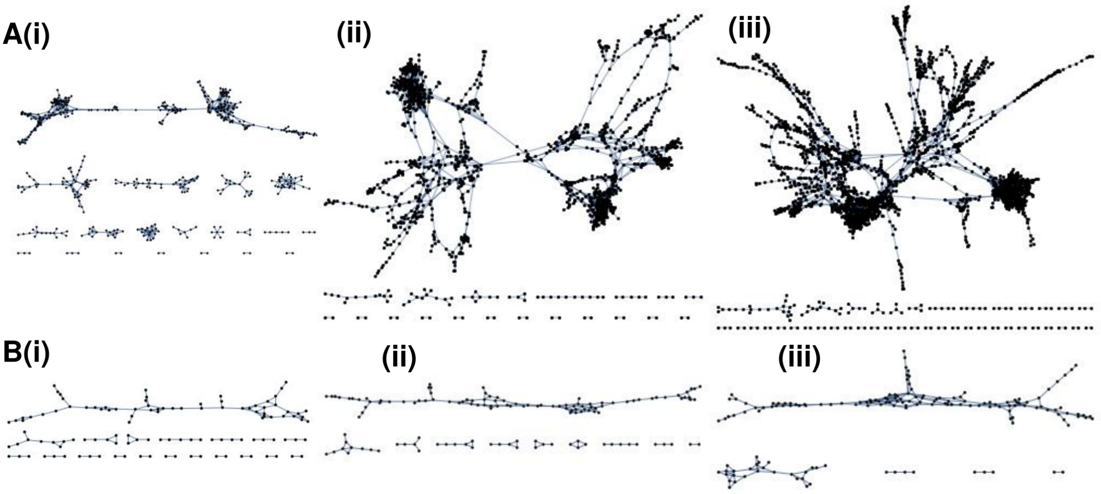


**C**

| | logMeanDegree (at 24 seconds) | InterMitoMean (at 24 seconds) | MeanSpeed (0–24 seconds) | DegreeCV (at 24 seconds) | Network Efficiency (at 24 seconds) |
|---|---|---|---|---|---|
| *Independent Video 1* | 1.34 | 1.86 | 4.84 | 1.15 | 0.0918 |
| *Independent Video 2* | 1.31 | 1.39 | 0.178 | 0.585 | 0.108 |

**Figure S1: Networks and summary statistics from two independent videos of mitochondrial motion**, related to Figures 2 and 5**.** (A) Encounter networks over time from independent video 1 and (B) independent video 2, with comparable timepoints of (i) 4 seconds; (ii) 12 seconds; (iii) 24 seconds; (C) summary statistics for both cases (singletons are omitted). Glossary: Degree, number of direct neighbours of each node; InterMitoMean, mean distance to nearest neighbouring mitochondrion (µm); MeanCH, mean convex hull area "swept out" by a trajectory ($\mu m^2$); Network Efficiency, average "closeness" (reciprocal of shortest path length) between pairs of nodes. Speed is in $\mu m\ s^{-1}$. See Methods (Summary statistics section) for full summary statistic definitions.

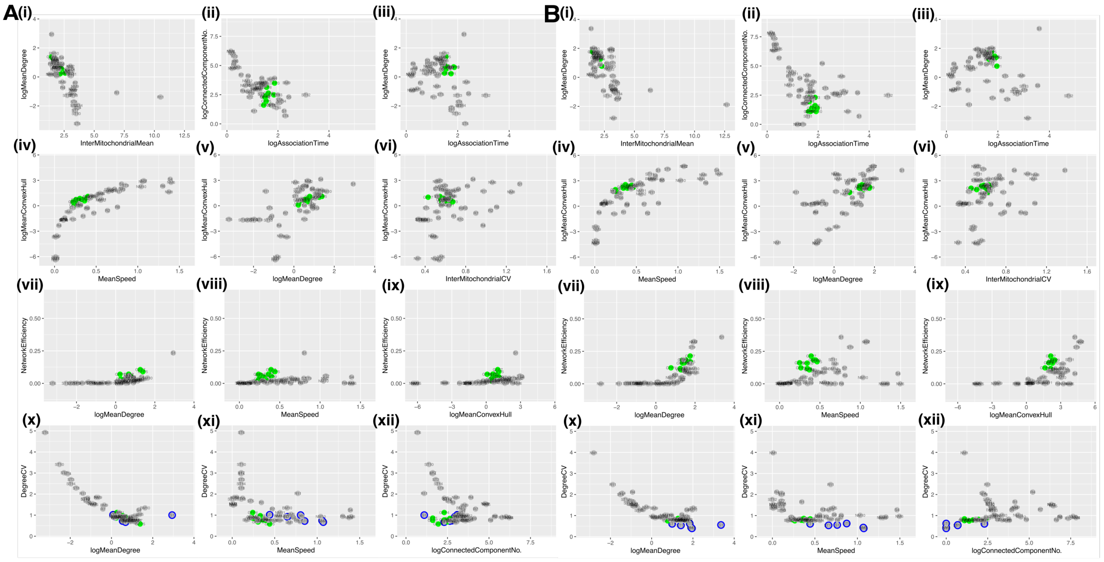


**Figure S2. Physical and social statistics at different timepoints**, related to Figure 5**.** (A), Statistics taken with the video end point at 1/10 total frame time (early time point, representative of 22 seconds). (B), Statistics taken with the video end point at 1/2 total frame time (early time point, representative of 115 seconds). Experimental results mtGFP ('GFP' green, n=10), theoretical results (grey, n=54), here name labels represent similarities in parameter grouping. Blue circles denote theoretical simulations of interest. CV = Coefficient of variation. Glossary: Degree, number of immediate neighbours of each node; InterMitoMean, Mean distance to nearest neighbouring mitochondrion (μm); MeanCH, mean convex hull area "swept out" by a trajectory ($\mu m^2$); Network Efficiency, average "closeness" (reciprocal of shortest path length) between pairs of nodes; Connected Components, number of connected subgraphs of the network; Association time, seconds spent in proximity to another agent/mitochondrion. Speed is in $\mu m\ s^{-1}$. See Methods (Summary statistics section) for full summary statistic definitions.

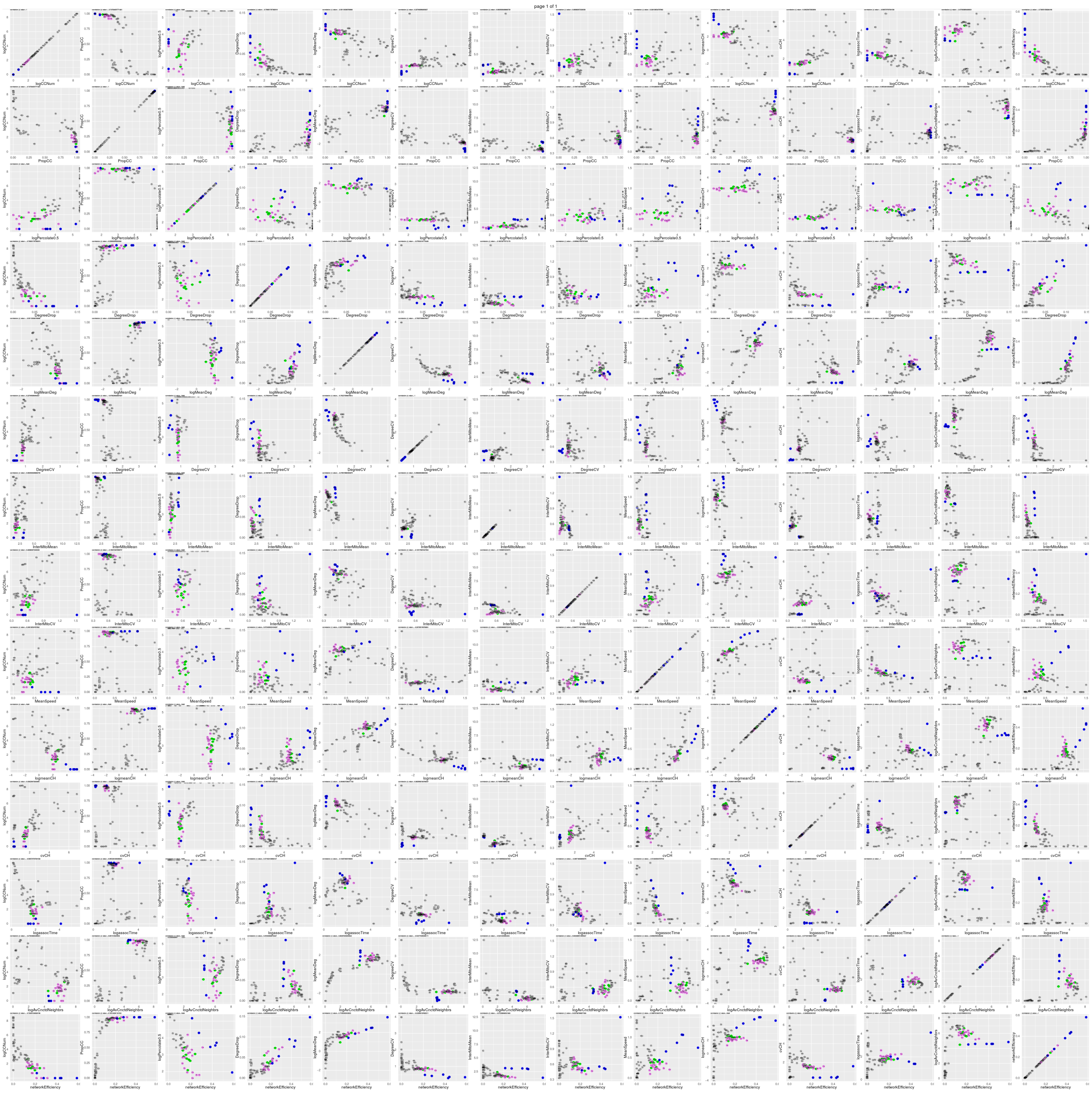

**Figure S3. All comparisons of physical and social statistics analysed,** related to Figures 5 and 6**.** Comparisons are taken with the video end point as the final frame (230 seconds). Experimental results mtGFP ('GFP' green, n=10) and *friendly* (violet, n=19), theoretical results (grey, n=54), here name labels represent similarities in parameter grouping. Blue circles denote theoretical simulations of interest. Axes label shorthand; CV = coefficient of variation, CH =Convex Hull, Deg = Degree, CC= connected component. See Methods (summary statistics and additional summary statistics sections) for full definitions.

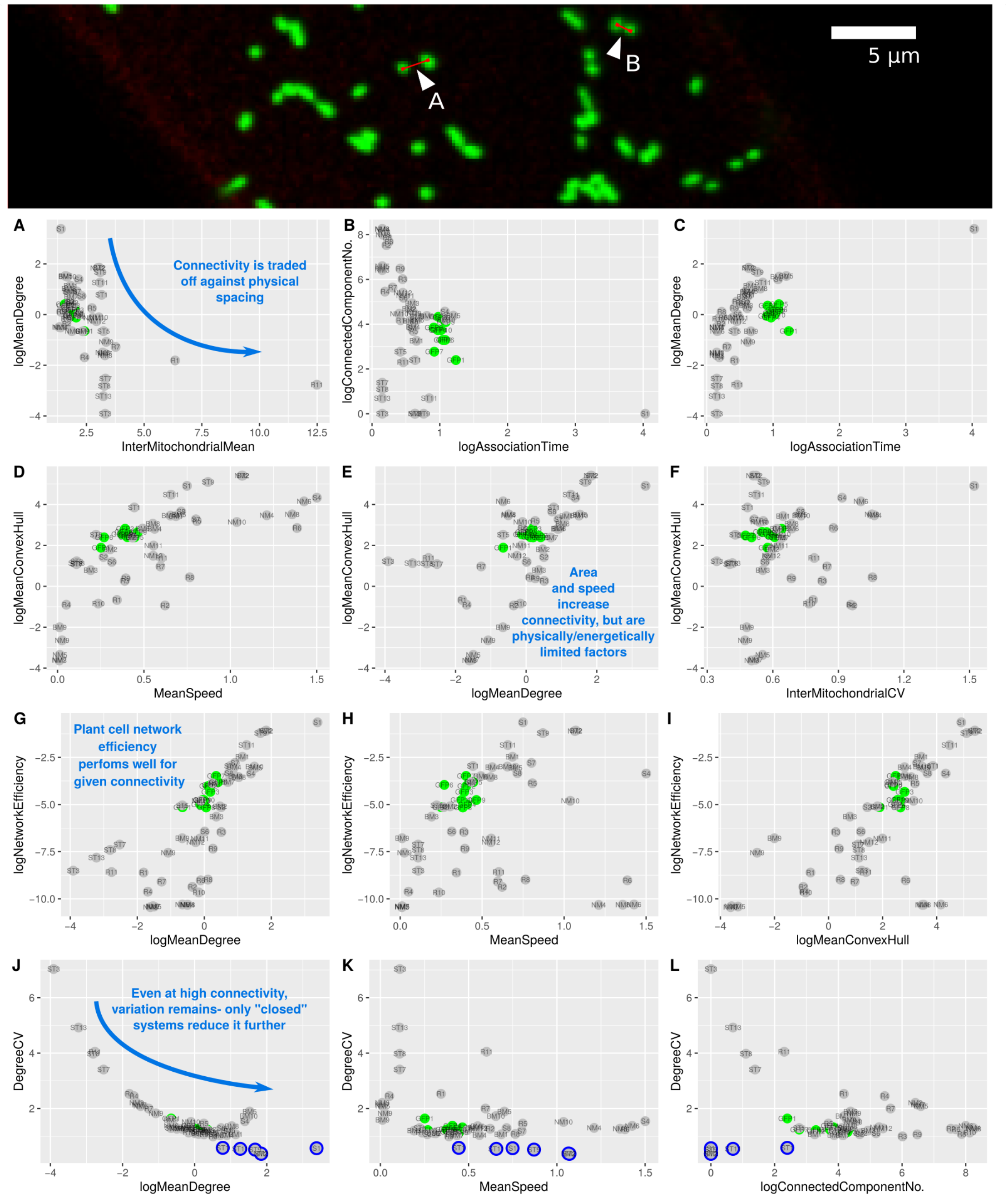


**Figure S4. Comparison of physical and social summary statistics between experimental and theoretical networks, enforcing adjacency for encounters between mitochondria**, related to Figure 5. Upper panel: confocal micrograph of mitochondria in *A. thaliana* hypoctyl cell (mitochondria (green), cell wall (red)) showing the original 1.6μm colocalisation distance (A, red line = 1.602μm in length), and the reduced 0.8μm colocalisation distance enforcing physical adjacency (B, red line = 0.805μm in length). Lower panels: Statistics plotted for experimental (green, *n* = 10) and simulated (grey, *n* = 47, different parameterisations) networks over 230 seconds. Labels of individual datapoints reflect individual experiments (GFP*n*, *n*=1-10, from single cell hypocotyl videos) and simulations (All described fully in Methods and full parameterisations shown in Table S1, except S5, NM1, ST4, ST6, ST10, ST12, ST14). Glossary: Degree, number of immediate neighbours of each

node; InterMitoMean, Mean distance to nearest neighbouring mitochondrion (μm); MeanCH, mean convex hull area "swept out" by a trajectory ($\mu m^2$); Network Efficiency, average "closeness" (reciprocal of shortest path length) between pairs of nodes; Connected Components, number of connected subgraphs of the network; Association time, seconds spent in proximity to another agent/mitochondrion. Speed is in $\mu m\ s^{-1}$. See Methods (Summary statistics section) for full summary statistic definitions.

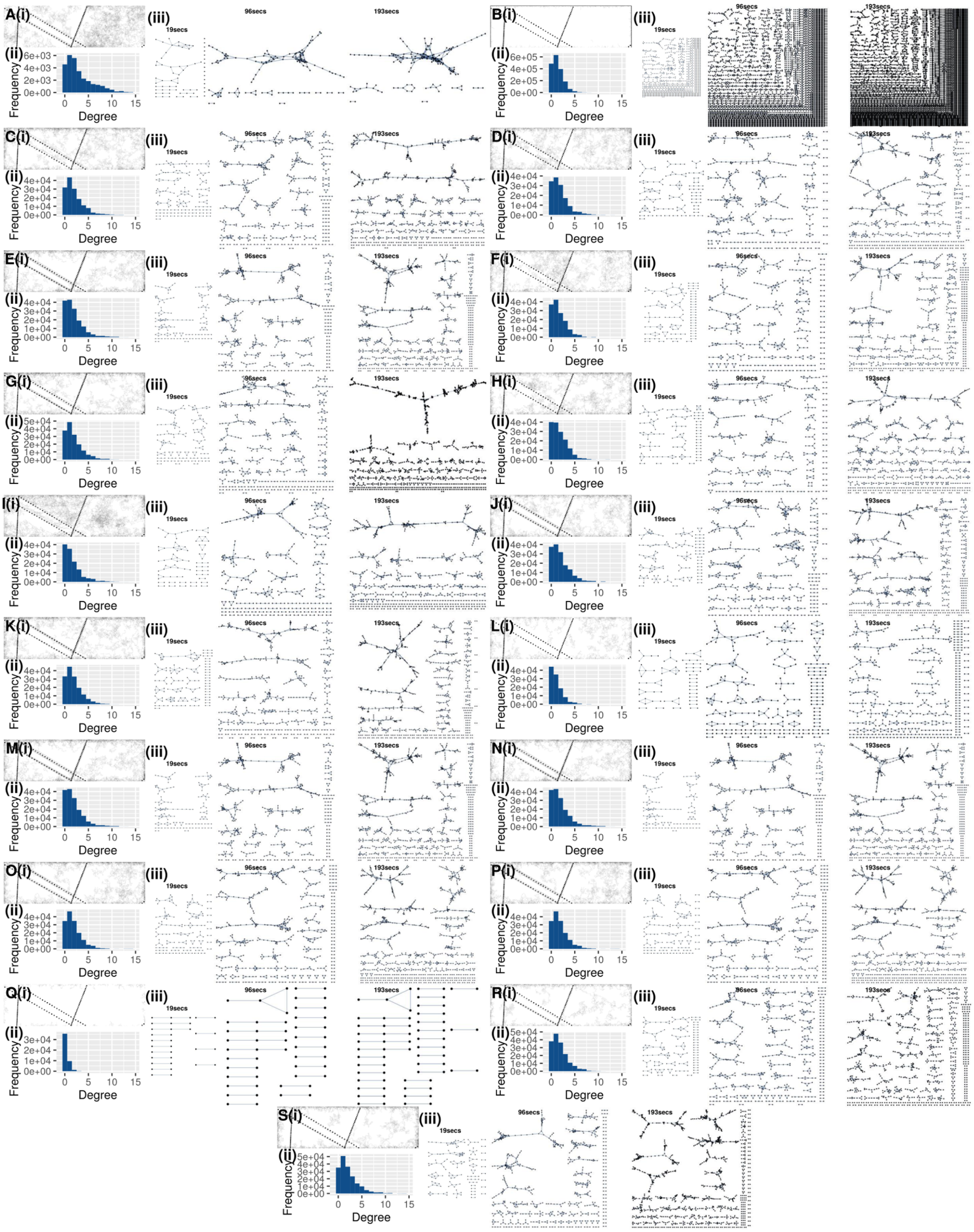

**Figure S5. Exploration of a range of simulation parameterisations showing the range of possible behaviours of agents in the cell and networks of encounters**, related to Figure 4**.** Models A-S represent 19 systems differing from an arbitrary default state**: A:** Low rate of agent loss at boundary region $K_{out} = 0.009\ s^{-1}$, **B:** High rate of agent loss at boundary region $K_{out} = 0.774\ s^{-1}$, **C:** 2:1 ratio of attachment to detachment of agents at cytoskeletal strands $K_{on} = 0.172\ s^{-1}$, $K_{off} = 0.086\ s^{-1}$, **D:** 1:2 ratio of attachment to detachment of agents at cytoskeletal strands $K_{on} = 0.086\ s^{-1}$, $K_{off} = 0.172\ s^{-1}$, **E:** 2:1 ratio of attachment to detachment of agents at cytoskeletal strands $K_{on} = 0.86\ s^{-1}$, $K_{off} = 0.43\ s^{-1}$, **F:** 1:2 ratio of attachment to detachment of agents at

cytoskeletal strands $K_{on}$ = 0.43 $s^{-1}$, $K_{off}$ = 0.86 $s^{-1}$, **G:** 5:1 ratio of attachment to detachment of agents at cytoskeletal strands $K_{on}$ = 0.86 $s^{-1}$, $K_{off}$ = 0.172 $s^{-1}$, **H:** 1:5 ratio of attachment to detachment of agents at cytoskeletal strands $K_{on}$ = 0.172 $s^{-1}$, $K_{off}$ = 0.86 $s^{-1}$, **I:** High hydrodynamic force $F_{hydro}$ = 0.990 **J:** Low hydrodynamic force $F_{hydro}$ = 0.010, **K:** Higher speed of agents on cytoskeletal strands $K_{cyt}$ = 0.85μm/s, **L:** Lower speed of agents on cytoskeletal strands $K_{cyt}$ = 0.009μm/s, **M:** High scaling of motion when mitochondria are interacting $K_{mito}$= 0.85 μm/s, but with scaling distance $d_{mito}$ = 0μm, **N:** Low scaling of motion when mitochondria are interacting $K_{mito}$= 0.009 μm/s, but with interaction range between mitochondria $d_{mito}$ = 0μm. **O:** Large interaction range $d_{mito}$=0.99μm, but with low scaling motion of interacting mitochondria $K_{mito}$= 0 μm/s, **P:** Small interaction range $d_{mito}$=0.01μm, but with low scaling motion of interacting mitochondria $K_{mito}$= 0 μm/s. **Q-S:** All have larger interaction range of $d_{mito}$ = 5μm, and a respective $K_{mito}$= 0, 0.2, 0.5 μm/s scaling motion of mitochondria. For each system, we show: **(i)** Traces of paths of model mitochondria over time within simulation space. Note completely static mitochondria do not appear on traces. Where present, cytoskeletal strands are shown as black dots. **(ii)** Degree distributions of the four systems, for all nodes over whole timeframe (230 seconds). **(iii)** Mitochondrial encounter networks of these four simulated systems, at simulation times corresponding to experimentally-determined networks in Figure 2E (singletons are omitted). Full parameterisations and summary statistics of all models can be seen in Table 2.

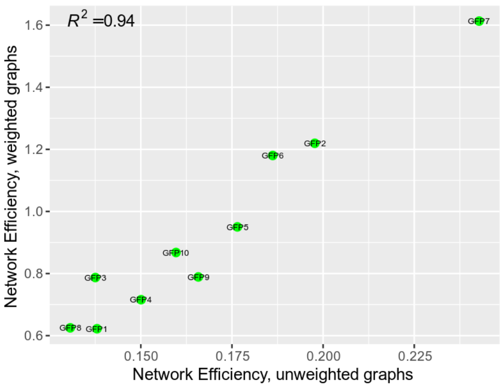


**Figure S6. Network efficiency of unweighted and weighted interaction networks**, related to Figure 5**.** Weighting of nodes is by 1/Association Time (secs), with the shortest distance between two nodes representative of a longer time spent associated. Data points are from the final frame of WT (GFP1-10) experimental time lapse videos. Network Efficiency; sum of all shortest routes across the network, See Methods (Summary statistics section) for full definition.

| Simulation Label | $N_{cyt}$ | $N_{mito}$ | $k_{out}$ | $d_{cyt}$ | $k_{on}$ | $k_{off}$ | $k_{cyt}$ | $d_{mito}$ | $k_{mito}$ | D | $F_{hydro}$ |
|---|---|---|---|---|---|---|---|---|---|---|---|
| **S1** | 4 | 128 | 0.000 | 6.008 | 0.809 | 0.479 | 6.412 | 8.339 | 0.770 | 0.754 | 0.000 |
| **S2** | 7 | 198 | 0.040 | 4.416 | 0.309 | 0.424 | 1.000 | 3.151 | 0.337 | 0.359 | 0.000 |
| **S4** | 16 | 123 | 0.098 | 4.860 | 0.945 | 0.234 | 1.300 | 0.252 | 0.620 | 0.851 | 0.526 |
| **S5** | 0 | 95 | 0.000 | 2.651 | 0.000 | 0.118 | 4.296 | 6.355 | 0.802 | 0.120 | 0.446 |
| **S6** | 1 | 154 | 0.050 | 7.079 | 0.000 | 0.706 | 9.341 | 5.627 | 0.408 | 0.431 | 0.000 |
| **S7** | 0 | 198 | 0.050 | 8.417 | 0.000 | 0.833 | 1.000 | 7.666 | 0.687 | 0.943 | 0.000 |
| **S8** | 12 | 124 | 0.056 | 1.630 | 0.441 | 0.343 | 5.014 | 3.176 | 0.490 | 0.441 | 0.000 |
| **NM1** | 0 | 100 | 0.000 | 0.000 | 0.000 | 0.000 | 0.000 | 0.000 | 0.000 | 0.010 | 0.000 |
| **NM2** | 0 | 100 | 0.000 | 0.000 | 0.000 | 0.000 | 0.000 | 0.000 | 0.000 | 0.990 | 0.000 |
| **NM3** | 0 | 100 | 1.000 | 0.000 | 0.000 | 0.000 | 0.000 | 0.000 | 0.000 | 0.010 | 0.000 |
| **NM4** | 0 | 100 | 1.000 | 0.000 | 0.000 | 0.000 | 0.000 | 0.000 | 0.000 | 0.990 | 0.000 |
| **NM5** | 0 | 100 | 1.000 | 0.000 | 0.000 | 0.000 | 0.000 | 0.000 | 0.000 | 0.010 | 0.990 |
| **NM6** | 0 | 100 | 1.000 | 0.000 | 0.000 | 0.000 | 0.000 | 0.000 | 0.000 | 0.990 | 0.990 |
| **NM7** | 0 | 100 | 1.000 | 0.000 | 0.000 | 0.000 | 0.000 | 0.000 | 0.000 | 0.010 | 0.010 |
| **NM8** | 0 | 100 | 1.000 | 0.000 | 0.000 | 0.000 | 0.000 | 0.000 | 0.000 | 0.990 | 0.010 |
| **NM9** | 0 | 100 | 0.100 | 0.000 | 0.000 | 0.000 | 0.000 | 0.000 | 0.000 | 0.010 | 0.000 |
| **NM10** | 0 | 100 | 0.100 | 0.000 | 0.000 | 0.000 | 0.000 | 0.000 | 0.000 | 0.990 | 0.000 |
| **NM11** | 5 | 100 | 0.100 | 1.000 | 1.000 | 0.500 | 1.000 | 0.000 | 0.000 | 0.174 | 0.000 |
| **NM12** | 0 | 100 | 0.100 | 0.000 | 0.000 | 0.000 | 0.000 | 0.000 | 0.000 | 0.174 | 0.000 |
| **BM1** | 7 | 198 | 0.040 | 4.416 | 0.309 | 0.424 | 1.000 | 3.151 | 0.337 | 1.000 | 0.000 |
| **BM2** | 7 | 198 | 0.040 | 4.416 | 0.309 | 0.424 | 1.000 | 3.151 | 0.337 | 0.400 | 0.000 |
| **BM3** | 7 | 198 | 0.040 | 4.416 | 0.309 | 0.424 | 1.000 | 3.151 | 0.337 | 0.200 | 0.000 |
| **BM4** | 7 | 198 | 0.040 | 4.416 | 0.309 | 0.424 | 1.000 | 3.151 | 0.337 | 0.800 | 0.000 |
| **BM5** | 7 | 198 | 0.040 | 4.416 | 0.309 | 0.424 | 1.000 | 3.151 | 0.337 | 0.600 | 0.800 |
| **BM6** | 7 | 198 | 0.040 | 4.416 | 0.309 | 0.424 | 1.000 | 3.151 | 0.337 | 0.600 | 0.200 |
| **BM7** | 7 | 198 | 0.040 | 4.416 | 0.309 | 0.424 | 1.000 | 3.151 | 0.337 | 0.600 | 0.000 |
| **BM8** | 7 | 198 | 0.040 | 4.416 | 0.309 | 0.424 | 1.000 | 3.151 | 0.337 | 0.600 | 0.400 |
| **BM9** | 7 | 198 | 0.040 | 4.416 | 0.309 | 0.424 | 1.000 | 3.151 | 0.337 | 0.000 | 0.000 |
| **BM10** | 7 | 198 | 0.040 | 4.416 | 0.309 | 0.424 | 1.000 | 3.151 | 0.337 | 0.600 | 0.600 |
| **ST1** | 5 | 100 | 0.000 | 0.000 | 0.000 | 0.000 | 1.000 | 0.000 | 1.000 | 0.400 | 0.000 |
| **ST2** | 5 | 100 | 0.000 | 0.000 | 0.000 | 0.000 | 1.000 | 0.000 | 1.000 | 1.000 | 0.000 |
| **ST3** | 0 | 100 | 0.000 | 0.000 | 0.000 | 0.000 | 1.000 | 0.000 | 1.000 | 0.100 | 0.000 |
| **ST4** | 15 | 100 | 0.000 | 0.000 | 0.000 | 0.000 | 1.000 | 0.000 | 1.000 | 0.100 | 0.000 |
| **ST5** | 5 | 100 | 0.000 | 0.000 | 0.000 | 0.000 | 1.000 | 0.000 | 1.000 | 0.200 | 0.000 |
| **ST6** | 20 | 100 | 0.000 | 0.000 | 0.000 | 0.000 | 1.000 | 0.000 | 1.000 | 0.100 | 0.000 |
| **ST7** | 2 | 100 | 0.000 | 0.000 | 0.000 | 0.000 | 1.000 | 0.000 | 1.000 | 0.100 | 0.000 |
| **ST8** | 6 | 100 | 0.000 | 0.000 | 0.000 | 0.000 | 1.000 | 0.000 | 1.000 | 0.100 | 0.000 |
| **ST9** | 5 | 100 | 0.000 | 0.000 | 0.000 | 0.000 | 1.000 | 0.000 | 1.000 | 0.800 | 0.000 |
| **ST10** | 4 | 100 | 0.000 | 0.000 | 0.000 | 0.000 | 1.000 | 0.000 | 1.000 | 0.100 | 0.000 |
| **ST11** | 5 | 100 | 0.000 | 0.000 | 0.000 | 0.000 | 1.000 | 0.000 | 1.000 | 0.600 | 0.000 |
| **ST12** | 5 | 100 | 0.000 | 0.000 | 0.000 | 0.000 | 1.000 | 0.000 | 1.000 | 0.000 | 0.000 |
| **ST13** | 8 | 100 | 0.000 | 0.000 | 0.000 | 0.000 | 1.000 | 0.000 | 1.000 | 0.100 | 0.000 |
| **ST14** | 10 | 100 | 0.000 | 0.000 | 0.000 | 0.000 | 1.000 | 0.000 | 1.000 | 0.100 | 0.000 |
| **R1** | 0 | 25 | 0.231 | 0.390 | 0.456 | 0.113 | 4.074 | 5.675 | 0.624 | 0.351 | 0.617 |
| **R2** | 1 | 92 | 0.585 | 0.363 | 0.685 | 0.040 | 1.945 | 5.062 | 0.417 | 0.820 | 0.537 |
| **R3** | 3 | 191 | 0.134 | 0.657 | 0.081 | 0.033 | 3.247 | 9.966 | 0.823 | 0.234 | 0.309 |
| **R4** | 2 | 90 | 0.718 | 0.637 | 0.712 | 0.796 | 9.271 | 5.810 | 0.044 | 0.318 | 0.136 |
| **R5** | 3 | 111 | 0.050 | 2.166 | 0.000 | 0.216 | 4.581 | 3.472 | 0.884 | 0.762 | 0.162 |
| **R6** | 13 | 159 | 0.565 | 0.796 | 0.985 | 0.366 | 4.181 | 4.870 | 0.879 | 0.413 | 0.642 |
| **R7** | 8 | 43 | 0.424 | 0.904 | 0.214 | 0.979 | 7.358 | 4.965 | 0.086 | 0.599 | 0.956 |
| **R8** | 3 | 161 | 0.526 | 0.340 | 0.440 | 0.735 | 3.929 | 0.422 | 0.423 | 0.673 | 0.411 |
| **R9** | 11 | 169 | 0.174 | 0.878 | 0.174 | 0.754 | 4.138 | 9.521 | 0.207 | 0.819 | 0.248 |
| **R10** | 0 | 183 | 0.745 | 0.313 | 0.259 | 0.964 | 4.211 | 6.884 | 0.571 | 0.177 | 0.609 |
| **R11** | 13 | 9 | 0.200 | 0.185 | 0.813 | 0.007 | 4.745 | 7.208 | 0.703 | 0.405 | 0.785 |

**Supplementary Table 1. Simulation parameterisations**, related to Figures 4,5, and S2-4. Simulation parameterisations used across Figure 4 and 5 and S2-4, with labels grouped by similarity. Parameter descriptions and units can be found in Materials and Methods. Rate units described here are per frame.

| Simulation Label | $N_{cyt}$ | $N_{mito}$ | $k_{out}$ | $d_{cyt}$ | $k_{on}$ | $k_{off}$ | $k_{cyt}$ | $d_{mito}$ | $k_{mito}$ | D | $F_{hydro}$ | logMean Degree (final frame) | InterMitoMean (overall) | logMean CH (overall) | Degree CV (final frame) | Network Efficiency (final frame) |
|---|---|---|---|---|---|---|---|---|---|---|---|---|---|---|---|---|
| **Model A** | 5 | 100 | 0.010 | 1.000 | 1.000 | 0.500 | 1.000 | 0.000 | 0.000 | 0.500 | 0.000 | 1.39 | 2.98 | 4.22 | 0.920 | 0.120 |
| **Model B** | 5 | 100 | 0.900 | 1.000 | 1.000 | 0.500 | 1.000 | 0.000 | 0.000 | 0.500 | 0.000 | 0.296 | 1.32 | -1.73 | 0.905 | 0.000101 |
| **Model C** | 5 | 100 | 0.100 | 1.000 | 0.200 | 0.100 | 1.000 | 0.000 | 0.000 | 0.500 | 0.000 | 0.697 | 2.89 | 1.76 | 0.964 | 0.00773 |
| **Model D** | 5 | 100 | 0.100 | 1.000 | 0.100 | 0.200 | 1.000 | 0.000 | 0.000 | 0.500 | 0.000 | 0.591 | 2.90 | 1.63 | 0.960 | 0.00662 |
| **Model E** | 5 | 100 | 0.100 | 1.000 | 1.000 | 0.500 | 1.000 | 0.000 | 0.000 | 0.500 | 0.000 | 0.669 | 2.77 | 1.99 | 1.01 | 0.00764 |
| **Model F** | 5 | 100 | 0.100 | 1.000 | 0.500 | 1.000 | 1.000 | 0.000 | 0.000 | 0.500 | 0.000 | 0.537 | 2.91 | 1.60 | 0.948 | 0.00447 |
| **Model G** | 5 | 100 | 0.100 | 1.000 | 1.000 | 0.200 | 1.000 | 0.000 | 0.000 | 0.500 | 0.000 | 0.699 | 2.86 | 1.82 | 0.955 | 0.00780 |
| **Model H** | 5 | 100 | 0.100 | 1.000 | 0.200 | 1.000 | 1.000 | 0.000 | 0.000 | 0.500 | 0.000 | 0.634 | 2.85 | 1.44 | 0.960 | 0.00557 |
| **Model I** | 5 | 100 | 0.100 | 1.000 | 1.000 | 0.500 | 1.000 | 0.000 | 0.000 | 0.500 | 0.990 | 0.529 | 2.66 | 2.62 | 1.13 | 0.00547 |
| **Model J** | 5 | 100 | 0.100 | 1.000 | 1.000 | 0.500 | 1.000 | 0.000 | 0.000 | 0.500 | 0.010 | 0.694 | 2.85 | 2.03 | 1.00 | 0.00666 |
| **Model K** | 5 | 100 | 0.100 | 1.000 | 1.000 | 0.500 | 0.990 | 0.000 | 0.000 | 0.500 | 0.000 | 0.666 | 2.85 | 1.93 | 0.901 | 0.00705 |
| **Model L** | 5 | 100 | 0.100 | 1.000 | 1.000 | 0.500 | 0.010 | 0.000 | 0.000 | 0.500 | 0.000 | 0.125 | 2.83 | 1.05 | 1.10 | 0.00222 |
| **Model M** | 5 | 100 | 0.100 | 1.000 | 1.000 | 0.500 | 1.000 | 0.000 | 0.990 | 0.500 | 0.000 | 0.669 | 2.77 | 1.99 | 1.01 | 0.00764 |
| **Model N** | 5 | 100 | 0.100 | 1.000 | 1.000 | 0.500 | 1.000 | 0.000 | 0.010 | 0.500 | 0.000 | 0.669 | 2.77 | 1.99 | 1.01 | 0.00764 |
| **Model O** | 5 | 100 | 0.100 | 1.000 | 1.000 | 0.500 | 1.000 | 0.990 | 0.000 | 0.500 | 0.000 | 0.626 | 2.73 | 1.84 | 0.945 | 0.00511 |
| **Model P** | 5 | 100 | 0.100 | 1.000 | 1.000 | 0.500 | 1.000 | 0.010 | 0.000 | 0.500 | 0.000 | 0.626 | 2.73 | 1.84 | 0.945 | 0.00511 |
| **Model Q** | 5 | 100 | 0.100 | 1.000 | 1.000 | 0.500 | 1.000 | 5.000 | 0.000 | 0.500 | 0.000 | -1.40 | 2.15 | -0.0812 | 2.00 | 0.000794 |
| **Model R** | 5 | 100 | 0.100 | 1.000 | 1.000 | 0.500 | 1.000 | 5.000 | 0.200 | 0.500 | 0.000 | 0.654 | 2.40 | 0.669 | 0.944 | 0.00335 |
| **Model S** | 5 | 100 | 0.100 | 1.000 | 1.000 | 0.500 | 1.000 | 5.000 | 0.500 | 0.500 | 0.000 | 0.791 | 2.50 | 0.895 | 0.932 | 0.00548 |

**Supplementary Table 2. Simulation parameterisations for parameter sweeps,** related to Figures 5 and S5. Simulation parameterisations for model exploration and influence of individual parameters (left), alongside physical and network summary statistics (right) taken from the network of the final frame, or overall from the time series (stated) for each system. Model traces, degree distributions and networks for each model can be seen in Figure S5.
