# Supplementary material for: Network analysis of Arabidopsis mitochondrial dynamics reveals a resolved tradeoff between physical distribution and social connectivity
Source: Cell Syst. 2021 May 19;12(5):419–431.e4. doi: 10.1016/j.cels.2021.04.006 (PMC8136767; doi:10.1016/j.cels.2021.04.006)
Supplement: Document S2. Transparent peer review records for DeTomaso et al [file mmc2.pdf]

Cell Systems

Transparent Peer Review Record

Network analysis of *Arabidopsis* mitochondrial dynamics reveals a resolved tradeoff between physical distribution and social connectivity

Joanna M. Chustecki, Daniel J. Gibbs, George W. Bassel, Iain G. Johnston

Summary

Initial Submission:	Received: Dec. 4, 2020
	Preprint: N/A
	Scientific editor: Quincey Justman, Ph.D.
First round of review:	Number of reviewers: Two
	Two confidential, zero signed
	Revision invited Jan. 13, 2021
	Major changes anticipated
	Revision received Feb. 22, 2021
Second round of review:	Number of reviewers: Two
	Two original, zero new
	Two confidential, zero signed
	Accepted Apr. 13, 2021
Data freely available:	Yes
Code freely available:	Yes

This Transparent Peer Review Record is not systematically proofread, type-set, or edited. Special characters, formatting, and equations may fail to render properly. Standard procedural text within the editor's letters has been deleted for the sake of brevity, but all official correspondence specific to the manuscript has been preserved.

Editorial decision letter with reviewers' comments, first round of review

Dear Iain,

Happy New Year! I'm enclosing the comments that reviewers made on your paper, which I hope you will find useful and constructive. As you'll see, they express interest in the study, but they also have a number of criticisms and suggestions. Based on these comments, it seems premature to proceed with the paper in its current form; however, if it's possible to address the concerns raised with additional experiments and/or analysis, we'd be interested in considering a revised version of the manuscript. ***We appreciate that the COVID-19 pandemic challenges and limits what you and your lab can do, so if you have concerns about the feasibility of revisions, let's schedule a Zoom call at our earliest mutual convenience.***

As a matter of principle, I usually only invite a revision when I'm reasonably certain that the authors' work will align with the reviewers' concerns and produce a publishable manuscript. In the case of this manuscript, the reviewers and I have two make-or-break concerns.

1. Reviewer 1 emphasizes that co-localization does not necessarily indicate that an interaction is functional. This is exceptionally important and must be reflected in the Revision. Precision and clarity within the text is a good first step, but what's really needed is an experimental demonstration that interaction is indeed functionally relevant. I appreciate that this may be challenging and I'm happy to talk about it over Zoom if that's helpful.
2. There is an important theme that runs through Reviewer 2's comments. It's this: can we be sure that apparent concordance between simulation and experiment is not the result of relatively trivial choices made about how to simulate? In the revision, we must be certain that this concordance is a result of deep concepts within the simulations, not, e.g. parameter choice or the choice to look at an arbitrary snapshot in time.

In addition to the concerns I've detailed above, I've highlighted portions of the reviews that strike me as particularly critical. I'd also like to be explicitly clear about an almost philosophical stance that we take at Cell Systems. We believe that understanding how models/simulations "fail" (or depart from biological behavior) is fundamentally interesting: it provides critical insight into understanding how they behave. Accordingly, when papers are transparent and forthright about the limitations and crucial contingencies of their approaches, we consider that to be a great strength, not a weakness. Please keep this in mind when addressing the Reviewers' concerns.

As you revise your manuscript, it's important that you and I stay on the same page. I'm always happy to talk, either over email or by phone, if you'd like feedback about whether your efforts are moving the manuscript in a productive direction. Do note that we generally consider papers through only one major round of revision, so the revised manuscript would be either accepted or rejected based on the next round of comments we receive from the reviewers. If you have any questions or concerns, please let me know. More technical information and advice about resubmission can be found below my signature. Please read it carefully, as it can save substantial time and effort later.

I look forward to seeing your revised manuscript.

All the best,
Quincey

Quincey Justman, Ph.D.
Editor-in-Chief, Cell Systems

Reviewers' comments:

Reviewer #1: The presented work is highly original and appropriate for cell biologists interested in quantitative analysis of sub-cellular dynamics.
To make the manuscript more accessible and to sharpen their arguments, I believe the authors should address the following major points:

1) In cell biology it is generally understood that co-localisation does not necessarily translate into interaction, while the presented work seems to use the two terms as exchangeable. The authors should carefully consider this distinction, and adapt their definitions and discussion of their results accordingly.
2) As a consequence of the above, the definitions of "physical" vs "social" "interaction" and their repeated use in the result interpretation is ambiguous and not very clear. If co-localisation is the only experimental measurement they have (and not molecular or physical interaction between organelles), the distinction between the two becomes really fuzzy. In absence of experimental data supporting true interaction (the ideal venue), ***[From QJ: As discussed above, we need experimental data that can unambiguously distinguish between physical (co-localization) and social (functional) interactions.]*** the authors should at least consider explaining more explicitly what they mean with the two terms, perhaps helping the reader with a simple diagram and/or example.
3) It is unclear to me the logical progression from Fig 4 to Fig 5: which of the four "systems" (A,B,C,D in Fig 4) is used to generate the simulated data in Fig 5? I understand that Fig 4 was presenting simulations corresponding to 4 set of possible parameters, but it is not clear which one is used in Fig 5
4) How are the experimental data (green) and simulated (gray) in Fig 5 compared? I was expecting some kind of "validation" of the model used, but it seems to me that you will need much more than N=10 experimental points to show that the simulated distributions shown in Fig 5 do indeed capture the experimental data. A proper statistical analysis would be required to show that the two distributions (experimental data and simulated points) are indeed indistinguishable.
5) When presenting the simulated statistics generated for the 4 sets of parameters (Fig 4), the authors should probably show how these compare with a trivial negative control such as random walk / Brownian motion. It would also be important to rule out that the observed (experimental) organelle dynamics is in fact not just Brownian motion.

As a minor but important point, I would also suggest the authors to revise some of their more convoluted and opaque statements, such as:
- lines 342-344
- lines 352-353

- lines 401-403
- lines 421-423 ("social interaction"? You only know they co-localise)
- lines 425-428 ("transfer of information"? Where is the evidence for that?)

Reviewer #2: The manuscript by Chustecki et al presents a new approach to formulating the interactions between discrete mitochondria as a `social network', highlighting the inherent tradeoff between spreading these organelles evenly throughout the cell interior and enabling frequent contact and exchange between individual organelles. While the main conclusion of the paper -- the fact that interactions between mitochondria require some amount of spatial clustering and heterogeneity -- is not particularly surprising, this work represents (to the best of our knowledge) the first quantitative analysis of such heterogeneity in live cells. The novel approach to analyzing experimental data is compelling, and the result that transport mutants ultimately converge to the same long-time interaction profile is particularly interesting. However, the modeling aspects of the paper could use some additional analysis and exposition. Specific concerns are listed below:

Major concerns:

1) The choice of parameters for the simplified model of mitochondrial motion seemed rather arbitrary and not wholly substantiated. It would be very helpful to provide additional exploration of the effect of each individual parameter and a discussion of why those particular parameters are the most relevant ones for determining how often mitochondria contact each other. Specific questions include:

a) Were kon/koff chosen arbitrarily or based on analysis of tracked mitochondrial trajectories? Similarly for the number of cytoskeletal filaments.

b) Do k_on and k_off matter as individual parameters or only in terms of their ratio (related to fraction of time spent in processive motion)?

c) Can mitochondria switch tracks at microtubule intersections to move in different directions?

d) How does the hydrodynamic coupling scale with distance? Is this handled through an Oseen tensor or by some other mechanism? Is it applied between mitochondria moving in different directions as well as ones on the same cytoplasmic filament? We were unable to find a detailed discussion in the methods of how the hydrodynamics were implemented.

e) Is the hydrodynamic coupling constant an important parameter, in that it substantially affects the results? Similarly for the k_mito coupling constant for adjacent mitochondria. Given the many parameters of the model, it would be helpful to see an analysis of whether anything additional is added to the final results by including these parameters as opposed to just varying the speed.

f) Why is the intermediate diffusivity value chosen in Fig. 4 (D=0.43um^2/s) so much higher than the observed diffusivities of mitochondria?

g) Do the simulation parameters matching the experimental data (in Fig. 5) make sense? Are all (or multiple) metrics matched for the same set of simulation parameters? Is it possible to extract some limits on the parameters from the comparison to experimental data?

2) The authors should provide additional discussion of the qualitative differences observed by altering mitochondrial motion through increased diffusion versus through attachment to cytoskeletal tracks. Fig 4E points to some differences (eg: high diffusion increases area covered by mitochondrial and network efficiency, as compared to rapid directed transport whereas the connectivity degrees are similar for both) but there is little direct discussion of whether and how the mode of motility matters.

3) The simulations treat k_out as an independent parameter separate from the diffusivity or transport rates of the mitochondria. Presumably the disappearance at the domain boundaries is associated with mitochondrial movement out of plane, and these transport parameters should be closely related to kout. The authors should discuss how the value of kout was selected given the specified motion parameters and why.

On a related note, the simulation results seem to exhibit piling up of mitochondria at the domain boundaries (Fig.1C; Fig. 4C,D) , presumably as a result of relatively slow rates of kout. This accumulation at the boundaries is not apparent in the experimental data. Could this discrepancy have a substantial influence on the quantitative results of the modeling? Specifically, one could imagine piling up organelles on a 1D boundary would lead to much higher frequency of interactions than if the same number of organelles was spread throughout the 2D interior.

4) The use of cumulative metrics for organelle interactions (eg: degree of interaction network over time) is somewhat concerning in that it is a metric very much dependent on the time scale of measurement. It is not clear how the simulations were initiated (with evenly spaced mitochondria?) or whether they were run over time-scales comparable to those required in order to reach steady-state. In other words, it is not clear whether the simulation results shown represent a transient period dependent on starting conditions or whether they correspond to some steady state distribution of organelle locations and interactions in the system. Would any of the results change if metrics were measured at longer time scales?

How does the timescale chosen compare to the time required for a mitochondrion to leave the domain (in experimental measurements)? Does the saturation observed in Fig. 2g correspond to the typical number of neighbors a mitochondrion hits before leaving the observed area? Are some trajectories truncated for other unspecified reasons? If so, there may be a bias to the measurements where the most motile mitochondria are likely to exhibit short trajectories before truncation, so that longer-time measurements of degree contain only the least motile agents.

5) It was not clear how mitochondria are introduced into the domain during the simulation. Do they re-enter through the periphery? Do they divide? Or is there no injection of new mitochondria so that the number in the domain decreases over time? If the latter, this would make the time-dependence of the measured parameters all the more problematic.

6) The authors should include more extensive discussion of the generalizability of these results to other cell types with different geometries of both the cell itself and the mitochondria.

7) The authors imply an inherent benefit to the cell from keeping the mitochondria evenly spaced throughout the interior. However, this benefit is not immediately obvious. In some cell types (eg: neurons) mitochondria are instead expected to cluster in regions of high metabolic demand. Is there evidence for spatial heterogeneity of metabolism in plant cells. For relatively small cells, would we expect the difference between evenly-spread vs clustered mitochondria to have any substantial effect on metabolism, given the rapid diffusivity of ATP?

8) Do the 'social networks' of mitochondrial contacts incorporate in any way multiple repeated contacts between the same pair of mitochondria? In principle, network edges could be assigned weights based on number of contacts or the total time in contact which could then impact the "efficiency" of the network for sharing material between discrete mitochondria.

Minor comments:
1) Figure axis labels such as "logmeanCH" should be written out in the figure itself or in the caption for improved readability.

2) In the simulations of "closed" systems, what do the mitochondria do when they hit the boundary? Reflect? Stop?

Authors' response to the reviewers' first round comments

Attached.

Editorial decision letter with reviewers' comments, second round of review

Dear Iain,

I'm very pleased to let you know that the reviews of your revised manuscript are back, the peer-review process is complete, and only a few minor, editorially-guided changes are needed to move forward towards publication.

In addition to the final comments from the reviewers, I've made some suggestions about your manuscript within the "Editorial Notes" section, below. Please consider my editorial suggestions carefully, ask any questions of me that you need, make all warranted changes, and then upload your final files into Editorial Manager. ***We hope to receive your files within 5 business days, but we recognize that the COVID-***

19 pandemic may challenge and limit what you can do. Please email me directly if this timing is a problem or you're facing extenuating circumstances.

As you look forward to acceptance, please do consider submitting one of the protocols you've developed in this paper to STAR Protocols, or extending this offer to one of your trainees. STAR Protocols is geared towards trainees and its key purpose is to provide complete and consistent instructions for how to conduct reproducible experiments. If you have any questions, please email starprotocols@cell.com.

I'm looking forward to going through these last steps with you. Although we ask that our editorially-guided changes be your primary focus for the moment, you may wish to consult our FAQ (final formatting checks tab) to make the final steps to publication go more smoothly. More technical information can be found below my signature, and please let me know if you have any questions.

All the best,
Quincey

Quincey Justman, Ph.D.
Editor-in-Chief, Cell Systems

Editorial Notes

Title: Your title is excellent, but I always ask authors to double-check that their titles are as effective as they can be. Note that an effective title is easily found on Pubmed and Google. A trick for thinking about titles is this: ask yourself, "How would I structure a Pubmed search to find this paper?" Put that search together and see whether it comes up is good "sister literature" for this work. If it does, feature the search terms in your title. You also may wish to consider that PubMed is sensitive to small differences in search terms.

Abstract: Your abstract is beautifully written, but it's significantly too long -- please limit yourself to 150 words, not including the peer review statement. Here is my first try, and note that if you can draw a more distinct connection between colocalization and mitochondrial function (in a cellular context), please do:

> *Mitochondria in plant cells largely as individual organelles which move, colocalise, and interact. Here, we represent these essential collective dynamics as a dynamic social network, describing the colocalisation of individuals over time. By combining single-cell live imaging of mitochondrial dynamics in the hypocotyl, individual-based modelling, and network analysis in wild-type Arabidopsis thaliana, and a mutant with compromised mitochondrial dynamics, we define an inevitable tradeoff between mitochondria's physical priorities (the even distribution of mitochondria through the cell) and "social" priorities (individuals interacting to facilitate exchange of chemicals and information). This tradeoff results in a tension between maintaining mitochondrial spacing and facilitating colocalisation. We find that plant cells resolve this tension to favour networks with high efficiency for exchange of XXX. We posit that combination of physical modelling coupled to*

experimental data through network analysis sheds light on the fundamental principles underlying these complex organelle dynamics.

Manuscript Text: As usual, your text is beautifully written. Thank you! A few notes:

- We favor slim Discussions that do not reiterate what's found in the Results beyond a brief transitional summary and are limited to around four medium sized paragraphs. Please consider deleting lines 563-612. I don't think they strengthen the paper and I'm worried that readers won't persist past this section to get to the discussion from line 613 onwards, which is excellent, indeed.
- When possible, consider breaking up long paragraphs. This is a minor style point, but shorter paragraphs are friendlier to read.
- House style disallows editorializing within the text (e.g. strikingly, surprisingly, importantly, etc.), especially the Results section. These terms are a distraction and they aren't needed—your excellent observations are certainly impactful enough to stand on their own. Please remove these words and others like them. “Notably” is suitably neutral to use once or twice if absolutely necessary.

Figures and Legends: Please ensure that your figure legends are defined operationally in the Legends (for example, what does Network Efficiency mean?), referring to the STAR Methods as necessary and making sure that units are indicated. Please revise your Figure Legends to make them fulsome and complete, with all variables/terms/units defined; if you are worried about text length limits, please email me. Please also ensure that the type within your Figures is large enough to read on a printed page. Main text figures are limited to a single page, but it's fine for Supplemental Figures to extend across multiple pages.

We are very grateful to the reviewers for their time and energy on our manuscript, and for the positive and constructive comments. We have attempted to address them individually below and believe the manuscript has been strengthened, and clarified, by their valuable feedback.

Reviewers' comments:
Reviewer #1: The presented work is highly original and appropriate for cell biologists interested in quantitative analysis of sub-cellular dynamics.
To make the manuscript more accessible and to sharpen their arguments, I believe the authors should address the following major points:

1) In cell biology it is generally understood that co-localisation does not necessarily translate into interaction, while the presented work seems to use the two terms as exchangeable. The authors should carefully consider this distinction, and adapt their definitions and discussion of their results accordingly.

2) As a consequence of the above, the definitions of "physical" vs "social" "interaction" and their repeated use in the result interpretation is ambiguous and not very clear. If co-localisation is the only experimental measurement they have (and not molecular or physical interaction between organelles), the distinction between the two becomes really fuzzy. In absence of experimental data supporting true interaction (the ideal venue), [From QJ: As discussed above, we need experimental data that can unambiguously distinguish between physical (co-localization) and social (functional) interactions.] the authors should at least consider explaining more explicitly what they mean with the two terms, perhaps helping the reader with a simple diagram and/or example.

Thanks to all for highlighting this issue with terminology and interpretation. Our phrasing of “social interaction” indeed caused some ambiguity in our claims and interpretation. To clarify – we are not looking directly at the functional consequences of organelle proximity, which has been explored in a body of previous work (with which we now connect). Rather, we consider proximity to be interesting for three reasons including these consequences. First, proximity is a requirement for fusion – one striking type of functional interaction – and thus reports an “upper bound” on the potential for fusion to occur. Although explicit fusion events are common on a cellular timescale, they are rare within a reasonable imaging timescale, and capturing enough to characterise the correspond collective dynamics would be impossible without stressing (and killing!) the cell. As such, we consider colocalisation as informative about the potential for fusion while remaining experimentally observable -- and now show (see below) that our results are robust to tightening this gap between colocalisation and physical adjacency.

Second, organelle proximity without explicit fusion itself has functional metabolic consequences, including in shaping metabolite flows and levels, and signalling. Third, mitochondrial proximity is a readout of the collective dynamics of organelles in the cell and hence a way of analysing these dynamics even without considering functional consequences.

The first two functional consequences are laid out in excellent existing work on mitochondrial dynamics, which we now connect to more directly. To address the

misinterpretation due to our phrasing, we have replaced “interaction” with “encounter” throughout the text when we are talking about physical proximity rather than functional consequences. Further, to provide a closer link between physical proximity and genuine interaction, we have performed additional analysis, requiring an “encounter” to constitute direct physical adjacency of two organelles, and show that our results remain robust when this tighter definition of proximity – where organelles are indeed physically interacting – is required.

3) It is unclear to me the logical progression from Fig 4 to Fig 5: which of the four "systems" (A,B,C,D in Fig 4) is used to generate the simulated data in Fig 5? I understand that Fig 4 was presenting simulations corresponding to 4 set of possible parameters, but it is not clear which one is used in Fig 5

We’ve clarified the link between Figs 4 and 5 in the captions and text – though have also included a substantial expansion of the model investigation in response to another reviewer’s comments. The picture is that Fig 4 represents some specific parameterisations to demonstrate fundamental physical behaviours of the model. New SI content (Supplementary Figure 8) explores the parameter space of the model, and consequent network behaviour, in rather more depth. Fig 5 then compares a broader set of parameterisations to the behaviour observed experimentally, including those in Fig 4, to compare the particular “solution” biology has evolved to the range of behaviours that would in principle be possible to accomplish with the physical system.

4) How are the experimental data (green) and simulated (gray) in Fig 5 compared? I was expecting some kind of "validation" of the model used, but it seems to me that you will need much more than N=10 experimental points to show that the simulated distributions shown in Fig 5 do indeed capture the experimental data. A proper statistical analysis would be required to show that the two distributions (experimental data and simulated points) are indeed indistinguishable.

We have clarified our approach in the text here. With this study it is not our intention to solve an inverse problem or perform inference to find, for example, the maximum likelihood parameterisation given our observations. While this an interesting question, our aim here is to build a general model that can capture not just the behaviour observed in our experiments but also the range of other behaviours if the physical mechanisms underlying the system were parameterised differently. In this sense we are addressing the forward problem – characterising how the model behaves under a range of different parameterisations in order to identify what “universal” principles apply regardless of parameterisation (for example, necessary tradeoffs in behaviour).

5) When presenting the simulated statistics generated for the 4 sets of parameters (Fig 4), the authors should probably show how these compare with a trivial negative control such as random walk / Brownian motion. It would also be important to rule out that the observed (experimental) organelle dynamics is in fact not just Brownian motion.

We apologise for the lack of clarity here – in fact some of parameterisations were chosen exactly to be such a diffusive negative control. We have expanded upon this and included further supplementary content to explore the behaviour of different reduced models.

As a minor but important point, I would also suggest the authors to revise some of their more convoluted and opaque statements, such as:
- lines 342-344
- lines 352-353
- lines 401-403
- lines 421-423 ("social interaction"? You only know they co-localise)
- lines 425-428 ("transfer of information"? Where is the evidence for that?)

Thanks for these suggestions. We have rephrased throughout the manuscript to address these and the previous points, and hope that the interpretation of our work is more transparent as a result.

Reviewer #2: The manuscript by Chustecki et al presents a new approach to formulating the interactions between discrete mitochondria as a `social network', highlighting the inherent tradeoff between spreading these organelles evenly throughout the cell interior and enabling frequent contact and exchange between individual organelles. While the main conclusion of the paper -- the fact that interactions between mitochondria require some amount of spatial clustering and heterogeneity -- is not particularly surprising, this work represents (to the best of our knowledge) the first quantitative analysis of such heterogeneity in live cells. The novel approach to analyzing experimental data is compelling, and the result that transport mutants ultimately converge to the same long-time interaction profile is particularly interesting. However, the modeling aspects of the paper could use some additional analysis and exposition. Specific concerns are listed below:

Major concerns:

1) The choice of parameters for the simplified model of mitochondrial motion seemed rather arbitrary and not wholly substantiated. It would be very helpful to provide additional exploration of the effect of each individual parameter and a discussion of why those particular parameters are the most relevant ones for determining how often mitochondria contact each other. Specific questions include:

Thanks for this suggestion. We have added supplementary content (particularly Supplementary Figure S8) exploring the behaviour of the model with respect to a broader range of parameterisations. We also discuss below some specific points that the reviewer raises.

a) Were kon/koff chosen arbitrarily or based on analysis of tracked mitochondrial trajectories? Similarly for the number of cytoskeletal filaments.

b) Do k_on and k_off matter as individual parameters or only in terms of their ratio (related to fraction of time spent in processive motion)?

c) Can mitochondria switch tracks at microtubule intersections to move in different directions?

The values of k_on and k_off that we initially chose to reflect plausible behaviour were based on an informal comparison of ballistic vs diffusive motion in our simulations and videos. We have now, as suggested, explored the model's dependence on these values in more depth and identified that indeed it is their ratio rather than absolute values that have the most effect. Mitochondria are indeed capable of switching tracks, but this requires an associated k_on event. We have added a brief outline of these points in the text and a broader parameter search of the model in Supplementary Information.

d) How does the hydrodynamic coupling scale with distance? Is this handled through an Oseen tensor or by some other mechanism? Is it applied between mitochondria moving in different directions as well as ones on the same cytoplasmic filament? We were unable to find a detailed discussion in the methods of how the hydrodynamics were implemented.

e) Is the hydrodynamic coupling constant an important parameter, in that it substantially affects the results? Similarly for the k_mito coupling constant for adjacent mitochondria. Given the many parameters of the model, it would be helpful to see an analysis of whether anything additional is added to the final results by including these parameters as opposed to just varying the speed.

The model hydrodynamic interaction we employ is a very simple model, not intended to solve true fluid dynamics equations. It consists simply of a velocity contribution from an averaging of neighbouring velocities scaled by reciprocal distance. It is applied to all mitochondria regardless of filament coupling. Unless the corresponding coefficient takes a rather high value, this interaction plays little role in determining model outcome. k_mito also exerts a rather weak influence on the overall behaviour. We have added a brief outline of these points in the text and a broader parameter search of the model in Supplementary Information.

f) Why is the intermediate diffusivity value chosen in Fig. 4 (D=0.43um^2/s) so much higher than the observed diffusivities of mitochondria?

Thanks for this point. We initially chose this value simply to reflect an intermediate case between the two extreme model versions we presented in Fig. 4. However, as the reviewer notes, there is no reason for this intermediate case to not involve a biologically plausible value. We have replaced this example with a value better matched to experimental observations.

g) Do the simulation parameters matching the experimental data (in Fig. 5) make sense? Are all (or multiple) metrics matched for the same set of simulation parameters? Is it possible to extract some limits on the parameters from the comparison to experimental data?

Here we are not explicitly attempting to find the best parameterisation to match biological observations, but rather to compare the observed biological behaviour to the range of behaviours observed under a range of model parameterisations. The behavioural trends from the range of parameterisations give us an idea of what "tradeoffs" the system fundamentally faces – which priorities cannot be simultaneously met under any parameterisation. The comparison to biological data then reveals which solutions to these theoretical tradeoffs have been identified and employed in biology. We have expanded on this approach in the text following this comment and a similar comment from the other reviewer.

2) The authors should provide additional discussion of the qualitative differences observed by altering mitochondrial motion through increased diffusion versus through attachment to cytoskeletal tracks. Fig 4E points to some differences (eg: high diffusion increases area covered by mitochondrial and network efficiency, as compared to rapid directed transport whereas the connectivity degrees are similar for both) but there is little direct discussion of whether and how the mode of motility matters.

We have edited and adapted the text associated with Fig 4 where we discuss this point, highlighting that the quasi-1D motion along strands facilitates more efficient connectivity increases than 2D diffusion.

3) The simulations treat k_out as an independent parameter separate from the diffusivity or transport rates of the mitochondria. Presumably the disappearance at the domain boundaries is associated with mitochondrial movement out of plane, and these transport parameters should be closely related to kout. The authors should discuss how the value of kout was selected given the specified motion parameters and why.

On a related note, the simulation results seem to exhibit piling up of mitochondria at the domain boundaries (Fig.1C; Fig. 4C,D) , presumably as a result of relatively slow rates of kout. This accumulation at the boundaries is not apparent in the experimental data. Could this discrepancy have a substantial influence on the quantitative results of the modeling? Specifically, one could imagine piling up organelles on a 1D boundary would lead to much higher frequency of interactions than if the same number of organelles was spread throughout the 2D interior.

Thanks for raising this point. Initial k_out values were chosen informally to match the number of trajectories observed in simulation and experiment (exit and entry of mitochondria correspond to termination and initiation of a trajectory, so the number of tracked trajectories is a function of k_out). Following this comment we investigated different values of k_out more thoroughly and found that model behaviour is preserved under a wide range of values; it is only as the situation gets rather extreme, and influx and efflux dominate the system, that the corresponding network behaviour changes as the

reviewer suggests due to “buildup”. We have added a brief outline of these points in the text and a broader parameter search of the model in Supplementary Information.

4) The use of cumulative metrics for organelle interactions (eg: degree of interaction network over time) is somewhat concerning in that it is a metric very much dependent on the time scale of measurement. It is not clear how the simulations were initiated (with evenly spaced mitochondria?) or whether they were run over time-scales comparable to those required in order to reach steady-state. In other words, it is not clear whether the simulation results shown represent a transient period dependent on starting conditions or whether they correspond to some steady state distribution of organelle locations and interactions in the system. Would any of the results change if metrics were measured at longer time scales?

Apologies for the absence of this important information. The simulations were indeed analysed after a burn-in period empirically determined to be sufficient for the initial conditions to be forgotten. Certainly results will change for longer-time measurements, as the results and the dynamic networks that underlie them are a function of the time window over which measurements are taken – we characterise this behaviour by taking three observation windows for our measurements of interest. But the burn-in period means that the simulation results are not dependent on particular initial conditions. We have added this description to the text.

How does the timescale chosen compare to the time required for a mitochondrion to leave the domain (in experimental measurements)? Does the saturation observed in Fig. 2g correspond to the typical number of neighbors a mitochondrion hits before leaving the observed area? Are some trajectories truncated for other unspecified reasons? If so, there may be a bias to the measurements where the most motile mitochondria are likely to exhibit short trajectories before truncation, so that longer-time measurements of degree contain only the least motile agents.

In both simulations and experiments, mitochondria do leave the domain during the timescale of the measurements. In experiments, some trajectories are truncated by the tracing process, as mitochondria move out of the plane of vision or are incorrectly matched between frames, although these effects are comparatively rare. Despite this, the effect that the reviewer mentions as bias may well be present, as rapidly moving mitochondria quickly reach the edge of the region of interest. However, we do not believe that this will lead to the case the reviewer subsequently mentions -- if these rapidly-moving mitochondria acquire high degrees, they will be "remembered" throughout the time course. This effect represents a dynamic feature of the system that is consistent across experiment and model, and means that in a sense the connectivities we report reflect a "lower bound" on the connectivities of particularly itinerant mitochondria through the time course -- we now discuss this point further in the text.

5) It was not clear how mitochondria are introduced into the domain during the simulation. Do they re-enter through the periphery? Do they divide? Or is there no injection of new

mitochondria so that the number in the domain decreases over time? If the latter, this would make the time-dependence of the measured parameters all the more problematic.

The mitochondria re-enter through the periphery, so that the efflux “loss” of mitochondria to a bath of other cellular regions is balanced by the influx “gain” of mitochondria from this bath. We have expanded upon this in the text.

6) The authors should include more extensive discussion of the generalizability of these results to other cell types with different geometries of both the cell itself and the mitochondria.

We have added discussion on these points -- including (thanks for provoking this thought!) the speculation that these principles may also shape mitochondrial structure in the reticulated networks found in other organisms.

7) The authors imply an inherent benefit to the cell from keeping the mitochondria evenly spaced throughout the interior. However, this benefit is not immediately obvious. In some cell types (eg: neurons) mitochondria are instead expected to cluster in regions of high metabolic demand. Is there evidence for spatial heterogeneity of metabolism in plant cells. For relatively small cells, would we expect the difference between evenly-spread vs clustered mitochondria to have any substantial effect on metabolism, given the rapid diffusivity of ATP?

Even spacing of mitochondria seems to be sufficiently beneficial to be consistently observed in small yeast cells, following the citations in the introduction, and in other situations where mitochondria are more spread through the cell than a random null model would imply, and where compromising this spread challenges the cell. We do agree that the mechanism(s) behind this advantage are far from resolved and do not propose a single hypothesis for this. Metabolism / ATP distribution may be one priority (even a fast-diffusion metabolite can still have substantial concentration gradients in the cell depending on the production and use rates of sources and sinks); facilitating colocalisation with other organelles may be another; rapid signalling may be another. We introduce these points in the text but do not (and would not!) commit to any particular primary mechanism without further investigation (a parallel strand of our research is geared towards this).

8) Do the 'social networks' of mitochondrial contacts incorporate in any way multiple repeated contacts between the same pair of mitochondria? In principle, network edges could be assigned weights based on number of contacts or the total time in contact which could then impact the "efficiency" of the network for sharing material between discrete mitochondria.

Thanks for this interesting suggestion! To explore this we tracked the association time between mitochondrial pairs and used the reciprocal of this association time to reweight the edges of our social network. We found a tight correlation between network efficiency computed from the unweighted and weighted networks, suggesting that association time and “raw” association structure are quite linked. This is included as new supplementary information but we will explore this connection further in future work.

Minor comments:
1) Figure axis labels such as "logmeanCH" should be written out in the figure itself or in the caption for improved readability.

We have edited figure captions according to make our shorthand clearer.

2) In the simulations of "closed" systems, what do the mitochondria do when they hit the boundary? Reflect? Stop?

In the closed case, steps that would cross the boundary are truncated at the boundary. We have clarified this in the text.
